# Natural climate solutions provide robust carbon mitigation capacity under future climate change scenarios

**DOI:** 10.1038/s41598-023-43118-6

**Published:** 2023-11-03

**Authors:** David C. Marvin, Benjamin M. Sleeter, D. Richard Cameron, Erik Nelson, Andrew J. Plantinga

**Affiliations:** 1 Salo Sciences, Inc., San Francisco, CA USA; 2 U.S. Geological Survey, Seattle, WA USA; 3 The Nature Conservancy, San Francisco, CA USA; 4 Bowdoin College, Brunswick, ME USA; 5 University of California, Santa Barbara, CA USA

**Keywords:** Climate-change ecology, Ecosystem ecology, Environmental economics

## Abstract

Natural climate solutions (NCS) are recognized as an important tool for governments to reduce greenhouse gas emissions and remove atmospheric carbon dioxide. Using California as a globally relevant reference, we evaluate the magnitude of biological climate mitigation potential from NCS starting in 2020 under four climate change scenarios. By mid-century NCS implementation leads to a large increase in net carbon stored, flipping the state from a net source to a net sink in two scenarios. Forest and conservation land management strategies make up 85% of all NCS emissions reductions by 2050, with agricultural strategies accounting for the remaining 15%. The most severe climate change impacts on ecosystem carbon materialize in the latter half of the century with three scenarios resulting in California ecosystems becoming a net source of carbon emissions under a baseline trajectory. However, NCS provide a strong attenuating effect, reducing land carbon emissions 41–54% by 2100 with total costs of deployment of 752–777 million USD annually through 2050. Rapid implementation of a portfolio of NCS interventions provides long-term investment in protecting ecosystem carbon in the face of climate change driven disturbances. This open-source, spatially-explicit framework can help evaluate risks to NCS carbon storage stability, implementation costs, and overall mitigation potential for NCS at jurisdictional scales.

## Introduction

Over 30 percent of human carbon emissions are sequestered annually through photosynthesis and subsequent carbon storage in terrestrial biomass and soils^1^. Conversion of terrestrial ecosystems to urban or agricultural land uses, and human-caused disturbances like forest harvest, result in emissions of carbon dioxide (CO_2_) through the removal of vegetation and disturbance of soil. Natural climate solutions (NCS, also known as Nature Based Solutions)—land conservation, restoration, and management practices and policies—are strategies intended to reduce emission of greenhouse gases or increase the biological sequestration of CO_2_. Increasingly, NCS are recognized as important climate change mitigation strategies at global^2^, national^3, 4^, and subnational^5–7^ scales.

After the direct and immediate reduction of emissions from fossil fuel and industrial activities, NCS provide many advantages over other climate mitigation strategies such as direct air capture. They are immediately deployable, relatively inexpensive^2, 3^, and many have a compounding effect where per-area sequestration rates grow over time. Perhaps most importantly, they provide multiple ecosystem services co-benefits, including improved biodiversity, water, soil, air quality, and resilience to climate impacts^2^.

A disadvantage of NCS is their potential vulnerability to reversal through human-, natural-, or climate-exacerbated disturbances^8, 9^. Evaluating the stability—or instability—of NCS carbon storage is  necessary before they can become an effective global approach to fight climate change. To date, studies of NCS mitigation potential do not explicitly account for disturbances like wildfire, mortality, and drought that can reverse the carbon emission mitigation benefits of NCS. Each of these drivers are likely to increase over time due to climate change^10–12^, with growing evidence these trends have already begun^13–15^. Even in situations where the stability of long-term carbon storage is at risk, NCS can act as temporary carbon storage when implemented with net-zero fossil fuel emissions to reduce or delay peak climate warming^16^.

To assist with evaluating the increasing NCS commitments of countries and states^17^, we develop a framework to investigate the mitigation efficacy and economics of NCS implementation at a jurisdictional scale, rather than assessing the climate risk and stability of individual NCS projects (as highlighted by ref.^9^). We evaluate a targeted subset of conservation and restoration NCS scenarios implemented in both agricultural and natural systems between 2020 and 2100 across the State of California—a globally relevant reference given its large carbon sink potential, biogeographic diversity, and increasing climate change-driven disturbances. Additionally, we include two scenarios where large-scale NCS implementation is delayed until 2030 and 2040 to assess the impact on long-term mitigation potential^18^. We track changes in statewide ecosystem carbon balance with and without NCS under four climate futures—four downscaled Global Climate Model (GCM) outputs (CanESM2, CNRM-CM5, HadGEM2-ES, MIROC5) under Representative Concentration Pathway (RCP) 4.5—using a fully-coupled stochastic state-and-transition simulation model with carbon stocks and flows^19, 20^. To keep computational demands reasonable, we chose to model a single RCP based on prior work^20^ that found the main driver of variance in future carbon flux is the choice of GCM and not RCP. By spatially tracking wildfire, mortality, and climate-driven vegetation growth rates, we explicitly account for potential reversals of NCS-related carbon storage. We additionally assess the economic impact of NCS deployment by estimating annual implementation costs for landowners and approximating the magnitude of gains and losses NCS implementation could cause in some of California’s major economic sectors. Our work improves on previous studies by modeling the impact of land management interventions combined with natural and anthropogenic disturbances in a spatially-explicit manner under a range of future climates, evaluating the effect of delayed implementation, and quantifying the economic costs and benefits. This scalable framework may provide policymakers, government agencies, and landowners anywhere with guidance on the magnitude, efficacy, and economics of nature-based solutions to mitigate climate change.

## Results

### Cumulative emissions reduction potential

**Figure 1:**
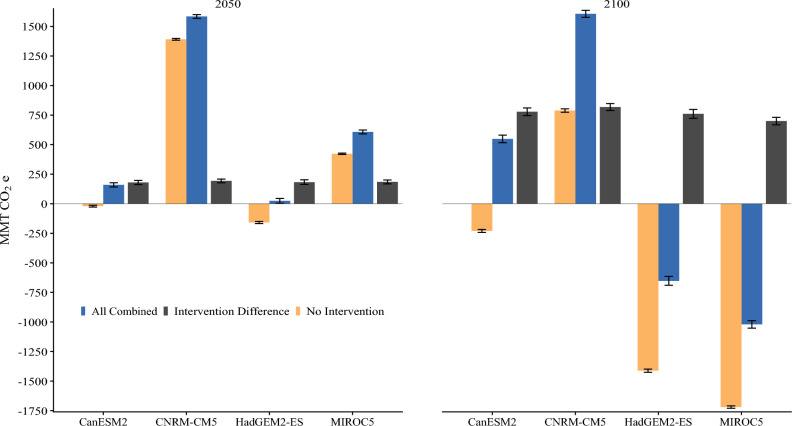
Cumulative net ecosystem carbon balance under each climate future. Mid- and end-of-century for all NCS interventions (blue) and no intervention (orange), with their difference in grey, relative to 2015. The four different climate futures (i.e., each GCM) used in the analysis are represented on the x-axis.

We use 2015 as the baseline reference year for total ecosystem carbon since this is the last year with empirical carbon and land use data in our model. Across all climate futures (i.e., the GCM-RCP combination), NCS interventions result in a net increase in carbon storage compared to taking no action. By 2050 large net carbon sinks—relative to 2015—are found in two climate futures even without any NCS interventions. Interventions in these two scenarios increased the sink by 14% to 1584 MMT CO_2_e under the CNRM-CM5 and by 44% to 607 MMT CO_2_e under MIROC5. In the other two scenarios, CanESM2 and HadGEM2-ES, the state’s lands flip from a net source (− 20 and − 158 MMT CO_2_e, respectively) to a net sink (160 and 25 MMT CO_2_e, respectively) with the addition of NCS interventions (Fig. 1). Reductions continue to accumulate for the remainder of the century, leading to cumulative additional carbon storage of between 699 and 818 MMT CO_2_e by 2100 when NCS interventions are implemented (Table 1).

**Table 1:** Cumulative emissions reduction potential from combined NCS intervention scenarios.

	Climate future			
	CanESM2	CNRM-CM5	HadGEM2-ES	MIROC5
Year	No intervention delay			
2050	180 (160, 199)	193 (177, 210)	183 (161, 205)	185 (164, 206)
2100	778 (740, 816)	818 (788, 848)	760 (714, 807)	699 (657, 742)
Year	10-year delay			
2050	113 (86, 139)	134 (110, 158)	117 (101, 133)	126 (99, 152)
2100	684 (643, 724)	740 (697, 784)	677 (649, 706)	623 (574, 671)
Year	20-year delay			
2050	87 (61, 113)	96 (71, 120)	96 (71, 122)	103 (72, 134)
2100	574 (527, 621)	656 (620, 692)	587 (535, 639)	545 (492, 598)

Net difference in mean cumulative emissions, in MMT CO_2_e, between no intervention and combined NCS intervention scenarios with lower and upper bounds in parenthesis for each climate future and intervention delay scenario.

**Figure 2:**
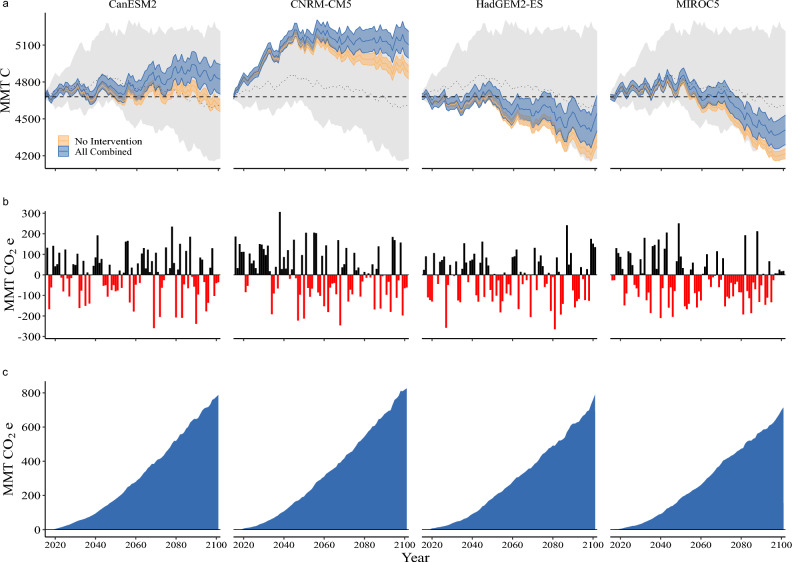
Total ecosystem carbon by climate future over the study period. Mean (solid line) and 95% confidence interval (colored shading) for no intervention (orange) and with NCS interventions (blue) with the mean (dashed line) and 95% confidence interval (grey shading) across all futures in units of carbon (**a**). Horizontal dashed line is the total ecosystem carbon in 2015 (baseline). Annual net ecosystem carbon balance in units of CO_2_e showing source (red bars) and sinks (black bars) over the study period for the no intervention scenario (**b**). Difference in total ecosystem carbon in units of CO_2_e between the NCS interventions and no intervention (**c**).

The most severe impacts of climate change on California land carbon are projected to occur after 2050^20^. Three out of four climate futures show moderate (CanESM2) to severe (HadGEM2-ES, MIROC5) declines in ecosystem carbon by 2100 compared to 2050 (Fig. 2a), even with substantial interannual variation (Fig. 2b). However, NCS interventions provide substantial mitigation of these projected carbon losses (Fig. 2c). Both the HadGEM2-ES and MIROC5 climate futures see major carbon declines by 2100, but NCS interventions retain an additional 760 (714 to 807) and 699 (657 to 742) MMT CO_2_e, respectively. Again, in the CanESM2 climate future California’s lands flip from a net carbon source (− 230 MMT CO2e) to an even larger net sink (548 MMT CO_2_e) in 2100 with interventions. In the absence of NCS interventions, almost half of the carbon built-up under the CNRM-CM5 climate future by 2050 (1391 MMT CO_2_e) is lost by 2100 resulting in net storage increase of 788 MMT CO_2_e. With NCS interventions an additional 818 MMT of CO_2_e are instead captured in California’s lands, resulting in an even larger sink of 1607 MMT CO_2_e by 2100.

#### Land management interventions

**Figure 3:**
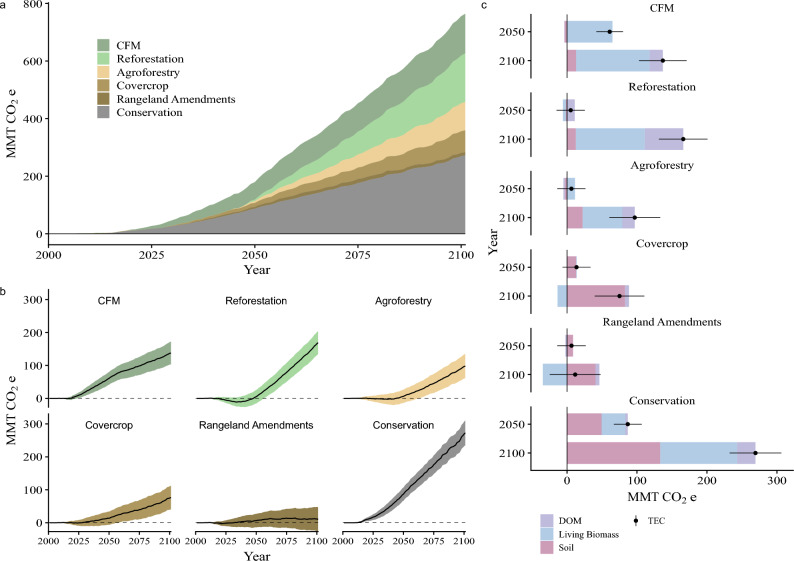
Effect of individual NCS interventions across all climate futures. Combined reduction potential as net change in total ecosystem carbon (TEC) relative to no intervention shown together (**a**) and individually with variation (shading) due to climate future and Monte Carlo iterations (**b**). Net change in carbon stocks by each major pool for each intervention relative to no intervention (**c**). DOM is dead organic material.

*Land conservation.* Preventing land conversion from natural to developed or agricultural use though conservation has the highest carbon storage potential of any single intervention, with, on average, an additional 84 (83 to 86) MMT CO_2_e by 2050 and 267 (265 to 269) MMT CO_2_e by 2100 (Fig. 3). Conservation represents 47% of total reduction potential by 2050, falling to 35% by 2100. While its relative contribution decreases by the end of the century, land conservation still provides 61% more reduction potential than reforestation, which is the second largest source of potential additional carbon storage. The carbon storage potential from land conservation is driven by a 55% reduction in agricultural conversion and 75% reduction in urbanization, which was determined by comparing a reduced LULCC (land use land cover change) change scenario against the business-as-usual scenario where historical rates of change were assumed to continue (for additional details see Supplemental Methods). See Table S1 for a breakdown of individual interventions by climate future.

#### Forest interventions

On average across climate futures the two forest-related interventions—changes to forest management (CFM) on lands used to produce timber and post-fire reforestation—collectively resulted in an additional 67 (51 to 71) MMT CO_2_e (mean and 95% Monte Carlo confidence intervals) stored in California ecosystems by 2050, rising to 304 (239 to 309) MMT CO_2_e by 2100 (Fig. 3). CFM contributes over 90% (62 MMT CO_2_e) of the total reductions from forest interventions in 2050, primarily because reforesting with seedlings/saplings takes a few decades to realize major carbon gains. But by 2100 reforestation increases substantially to 55% (167 MMT CO_2_e) of total reductions from forest interventions. While CFM has an immediate and substantial positive increase in reduction potential, reforestation takes another 35 years to surpass annual reductions of 5 MMT CO_2_e. Overall, forest interventions contribute 38% of the total reductions by 2050 and 40% by 2100.

#### Agricultural interventions

On average across climate futures, the three agricultural-related interventions—cover cropping, agroforestry, and rangeland amendments—contribute 27 (24 to 31) MMT CO_2_e in additional carbon stored by 2050, increasing to 184 (180 to 189) MMT CO_2_e by 2100 (Fig. 3). Cover cropping makes up over 50% (14 MMT CO_2_e) of the total by mid-century, with rangeland amendments and agroforestry each contributing approximately one-quarter of the agricultural intervention total. By the end of the century, additional carbon stored through agroforestry increased substantially to 52% (97 MMT CO_2_e), with cover cropping making up another 41% (75 MMT CO_2_e). Rangeland soil amendments, while doubling net cumulative carbon stored compared to 2050 (to 13 MMT CO_2_e), do not make a substantial contribution to the total agricultural reduction potential largely due to the relative loss of live biomass that would have accumulated in rangelands converted to perennial agriculture under the no intervention scenario (Fig. 3c). Overall, agricultural interventions contribute 15% and 24% of total potential reductions by 2050 and 2100, respectively.

### Economic evaluation of NCS interventions

Direct NCS intervention costs are substantial. Implementing all six land management interventions (combined NCS intervention scenario) would force landowners to forgo 5.9 billion USD (all USD values are measured in year 2017 USD) in present value terms over a 30 year implementation period (2020 to 2050). Direct costs refer to implementing and maintaining each intervention, such as planting and plowing under a cover crop each year. On an annualized basis, these interventions cost 382 million USD per year. We do not assess direct implementation costs beyond 2050 given the factors involved in estimating long-term economic costs of NCS interventions 30 to 80 years into the future would require an assessment outside the scope of this investigation.

Relative to taking no action, we estimate that the combined NCS intervention scenario would force landowners to incur incidental or indirect net costs ranging from 5.7 to 6.1 billion USD across the four climate futures from 2020 to 2050 (Table 2). This indirect net economic loss is entirely driven by reductions in the value of agricultural and developed land production resulting from the land conservation intervention, which outweigh increases in the value of the state’s managed forest industry (49–121 million USD for clearcut, 33–64 million USD for selection) and *reductions in the damage* caused by nitrogen use (7–12 million USD) (Table 2). We do not assess indirect costs beyond 2050 given the assessment of global agricultural and timber and local land markets 30 to 80 years into the future is beyond the scope of this investigation.

**Table 2:** Economic costs and benefits from 2020 NCS scenario.

	Climate future			
	CanESM2	CNRM-CM5	HadGEM2-ES	MIROC5
Direct cost				
Total	5872	5850	5879	5876
Annualized	382	381	382	382
Indirect cost				
Agriculture	5048	5344	4959	5036
* SCN*	− *7*	*3*	− *11*	− *12*
Developed	838	863	850	804
Forest Harvest	− 126	− 113	− 131	− 153
* Clearcut*	− *76*	− *49*	− *89*	− *121*
* Selection*	− *50*	− *64*	− *42*	− *33*
Total	5760	6094	5678	5686
Annualized	375	396	369	370
Direct + indirect costs				
Total	11,632	11,944	11,557	11,562
Annualized	757	777	752	752
Carbon benefit				
Total	1447	1528	1361	1408
Annualized	94	99	89	92

Estimated direct and indirect costs and global public good benefit generated by NCS interventions relative to the no intervention scenario over the period 2020–2050. Agricultural land value is inclusive of the social cost of nitrogen (SCN). Forest harvest is the sum of the clearcut and selection harvest values. Values are constant (2017) million USD and we use a 5% per annum rate to discount costs borne by California landowners and carbon benefits enjoyed across the globe. See Table S4 for economic results when using a 2.5% per annum rate to discount carbon benefits (but still a 5% per annum rate to discount costs).

After summing the direct and indirect costs generated by NCS interventions, California landowners face net intervention costs of 11.6–11.9 billion USD, depending on the climate future (Table 2). This translates to an annualized cost of 752–777 million USD. When accounting for both direct and indirect costs, the suite of NCS interventions cost between 62 and 65 USD per metric ton of CO_2_e. All cost calculations assume a 5% per annum discount rate on the assumption that costs of interventions that target landowners should be discounted using rates that reflect rates of return in capital markets (e.g., 5% per annum or more). Economic costs using the minimum and maximum assumptions are shown in Table S2 and Table S3, respectively.

For comparison, the direct economic benefit of the combined NCS intervention scenario, the monetary value of the additional carbon stored, is 1.4–1.5 billion USD when using a 5% discount rate (Table 2) and 9.6–10.5 billion USD when using a 2.5% discount rate (Table S4). Unlike the costs of NCS interventions, which are borne entirely by California landowners, the direct economic benefits of NCS intervention accrue to the whole world. Therefore, a lower per annum discount rate for benefits (e.g., 2.5% vs 5%) may be warranted. Economic benefits using the minimum and maximum assumptions and assuming a 5% discount rate are shown in Table S2 and Table S3, respectively.

Just two of six interventions are responsible for almost all of the indirect (land conservation) and direct cost (cover crop) borne by California landowners, with 94% of combined costs tied to these two interventions (Tables 3 and S5). Forest-based interventions have the lowest total cost (direct plus indirect) per ton of CO_2_e sequestered, with CFM actually generating additional value. Due to the relatively low amount of total carbon it sequesters combined with its very high direct cost, cover cropping’s cost to California landowners is often more than an order of magnitude higher on a per ton basis than other interventions.

**Table 3:** Cost and benefit per ton of CO_2_e for individual and combined interventions.

Intervention	Climate future											
	CanESM2			CNRM-CM5			HadGEM2-ES			MIROC5		
	Cost		Benefit	Cost		Benefit	Cost		Benefit	Cost		Benefit
	Direct	Indirect		Direct	Indirect		Direct	Indirect		Direct	Indirect	
Reforestation	36.06	–	4.87	no seq	–	no seq	94.87	–	− 14.65	46.02	–	2.39
CFM	–	− 2.12	8.34	–	− 1.93	8.42	–	− 2.08	7.67	–	− 2.35	8.42
Agroforestry	5.78	–	5.83	no seq	–	no seq	no seq	–	no seq	3.82	–	6.33
Cover Crop	710.15	–	14.13	1,335.09	–	9.09	403.01	–	2.39	162.33	–	9.02
Soil Amend	38.82	–	15.69	no seq	–	no seq	16.35	–	7.17	32.13	–	6.71
Conservation	–	69.86	7.75	–	77.00	7.39	–	71.78	7.22	–	63.75	7.64
Combined	32.64	32.02	8.05	30.27	31.53	7.91	32.14	31.04	7.44	31.79	30.76	7.62

Direct and indirect costs and global public good benefit per ton of CO_2_e sequestered relative to the no intervention scenario over the period 2020–2050. Interventions marked “no seq” means no net carbon storage under that climate future. Aggregate costs and benefits for each intervention are shown in Table S7. Dashes indicate no cost associated with the intervention. Values are constant (2017) USD and we use a 5% per annum rate to discount costs borne by California landowners and carbon benefits enjoyed across the globe. See Table S5 for economic results when using a 2.5% per annum rate to discount carbon benefits (but still a 5% per annum rate to discount costs).

A 10-year delay in implementation of the interventions reduces total costs (direct plus indirect) by about one-half across all of the climate futures (Table S6). Direct intervention cost is far lower due to 10 fewer years of implementation and the discounting of future costs, and losses in the value of agricultural production are about 30 percent as high. A 20-year delay results in relatively little carbon sequestration value accrued across the four climate futures, but similar total economic cost to the 10-year delay scenario.

## Discussion

From a carbon sequestration perspective, the aggregate NCS reduction potential of a suite of interventions is remarkably consistent and stable across climate futures, with NCS interventions storing between 180 and 193 additional MMT CO_2_e by 2050 compared to taking no additional action (Fig. 2). Even with substantial interannual variation in carbon dynamics driven by climate variability and natural and anthropogenic disturbances, we find NCS interventions result in persistent and growing additional carbon stores through the end of the century (Fig. 2c). This relative increase in carbon sequestration and emissions reductions from natural and agricultural lands provides a major economic benefit to the world (valued as high as $1.5 billion; Table 2). By 2050, two of four climate futures suggest the state’s lands will be a net carbon sink under a business as usual land management trajectory, storing anywhere from 422 to 1391 MMT more CO_2_e than the 2015 baseline. NCS interventions add an additional 185 to 193 MMT CO_2_e, increasing the land sink by up to 44%. The other two climate futures suggest the state will be a net source of carbon emissions, releasing a cumulative 20 to 158 MMT CO_2_e over the 2015–2050 time period. By implementing NCS interventions the state’s lands shift to a net carbon sink, instead storing a cumulative 24 to 160 MMT CO_2_e over 2015–2050.

Historically, California natural and working lands are a net annual source of carbon to the atmosphere^20, 21^. In previous work, we estimated the size of the net source at − 49.5 MMT CO_2_e/yr between 2011 and 2016^20^. However, there was considerable annual variability, primarily due to variations in climatic conditions, with the flux ranging from 220.6 (net sink) to − 329.6 (net source) MMT CO_2_e/yr. During this period, net carbon emissions from California's natural and working lands was equivalent to approximately 11% of the state's average annual total greenhouse gas emissions. Three studies of California’s future mitigation potential present relevant comparisons to our results, although differences in implementation scenarios, baseline LULCC assumptions, and incorporation of climate change impacts make direct comparisons difficult. In contrast to this study, Cameron et al.^5^ found among 14 interventions that the alternative land management and restoration activities, especially changes to the frequency of private land timber harvest, have a much larger mitigation potential by 2050 than avoided conversion of forest and grassland ecosystems. More limited definition of land conversion and lack of incorporation of soil carbon losses due to conversion explain some of the differences to the current study. Using the CALAND model, two studies looked at the potential for multiple land interventions to reduce emissions from disturbance, LULCC, and management activities^22^ and additionally under two climate scenarios^23^. These studies highlight the near-term trade-offs associated with advancing mitigation and adaptation goals simultaneously. In particular, the carbon cost of managing forests to reduce wildfire risk through increased biomass removal increases emissions in the near term (to 2030) but in combination with other land-based activities, can achieve GHG reductions. Simmonds et al.^23^ generates a similar estimate of cumulative GHG reductions (mean of 743 MMT CO_2_e; CanESM2 climate model, RCP 4.5) by 2100 to this study for the same climate future (778 MMT CO_2_e). Though not directly comparable with this study, collectively they assess a comprehensive set of land interventions that can support climate mitigation targets. Several studies have looked beyond California, assessing the mitigation potential of U.S. forest and agriculture sectors under various land management and restoration scenarios^3, 24–27^.

The economic analysis reveals the direct cost of all NCS interventions to California landowners is approximately 382 million USD annually under all climate futures. This represents about 8% of the annual funding for agricultural conservation programs recently made available under the Inflation Reduction Act of 2022^28^. Our economic model finds interventions generate indirect costs across California’s agricultural and developed land sectors of approximately the same magnitude as the direct cost (assuming immediate implementation). These indirect costs are driven by the relative loss of land in agricultural and developed uses when land conservation measures are implemented. However, modeled agricultural indirect costs due to NCS interventions are small relative to the total value of agricultural production in California: losses are just 0.65% of the net present value of agricultural net returns generated over the next 30 years from all agricultural land^29^, conservatively assuming no change in future net returns. Moreover, NCS interventions, including land conservation, generate many economically valuable co-benefits in addition to carbon sequestration^30^ that we do not measure in this research. Delaying implementation of NCS substantially reduces costs, but it also shrinks the value of carbon sequestered.

When viewed on a net cost-per-ton of CO_2_e basis, the costs of immediate implementation ($62–$65/tCO_2_e) are higher than current prices in carbon markets, but below those required to meet temperature goals in the Paris Agreement^31^ and far below direct air capture cost ($220/tCO_2_e)^32^ and the optimal carbon tax rate recently floated by the U.S. EPA ($190/tCO_2_e)^33^. Considering the latter estimate, if a $190/tCO_2_e tax were ever implemented, investment in the strategies analyzed here would represent significant cost savings for polluting firms (assuming a firm could offset an omitted tCO_2_e with a California NCS credit).

In comparison to the existing programs funded by the California Climate Investments Program, the strategies analyzed here are comparable or more expensive on average compared to other land based programs ($11/tCO_2_e for agricultural easements, $32/tCO_2_e for wetland and watershed restoration, $43/tCO_2_e for forest health as of 2019) but much lower than other programs ($173/tCO_2_e for Healthy Soils, $142/tCO_2_e urban forestry, $117/tCO_2_e for clean vehicle rebates as of 2019). Cost-per ton CO_2_e drops dramatically to $4–7 when considering only tree-based NCS interventions (CFM, reforestation, agroforestry). When combined, these three strategies represent almost as much carbon mitigation potential—under the CanESM2 and MIROC5 climate futures—as land conservation but come at less than 10% of the cost.

From the perspective of California’s effort to reach emissions neutrality by 2045^34^, NCS interventions provide a path to partially fill any emissions gap. Assuming the state can meet its 2030 emissions reductions target and then reduce emissions consistent with its 2050 target (emissions 80% below 1990 levels), approximately 1168 MMT of additional CO_2_e reductions are needed to reach carbon neutrality by 2045. NCS interventions starting immediately can provide as much as 17% of the reductions needed to meet that gap. Any implementation delay significantly reduces the amount that NCS can contribute and must come from steeper emissions cuts or more expensive removal technologies (Supplementary Results).

However, a mid-century focus misses the severe and persistent declines in land carbon during the latter half of the century in three of four climate futures (see also ref.^35^). Even with these severe climate-driven carbon losses, NCS interventions help to mitigate the effects of the decline, in one case by as much as 54% (HadGEM2-ES). Even in a generally warm-wet climate future where large amounts of carbon are stored by 2050 but halved in the latter half of the century (CNRM-CM5), NCS interventions are effective at mitigating potential losses. NCS interventions not only maintain the sink strength from 2050 but increase it by 17% through 2100.

Importantly, deploying a diverse portfolio of NCS can hedge against any single land management intervention underperforming due to unanticipated climate, land use, or economic reversals. A diverse set of NCS can target multiple carbon pools at the jurisdictional level that are ecologically, geographically, and temporally stratified. This is best illustrated by reforestation, which takes a few decades to begin accumulating carbon and eventually becomes the second largest mitigation strategy behind land conservation. Additionally, while aboveground living biomass from forest-based NCS represents the single largest carbon mitigation capacity, soil carbon plays an important role in nearly all NCS interventions (Fig. 1). Surprisingly, soil carbon storage from land conservation alone represents more than all agricultural interventions combined in 2050. This highlights the protection of existing carbon stocks as a major NCS solution.

Our study includes a few limitations that are important to consider. First, we did not model scenarios that included multiple RCPs. While the choice of RCP is an important factor in the magnitude and timing of atmospheric carbon dioxide levels, our previous work that included both RCP 4.5 and RCP 8.5 found far more variance in future carbon flux as a result of the choice of GCM (see Figs. 5 and 6 in ref.^20^). Second, we chose a subset of the dozens of possible natural and agricultural land management interventions. We modeled those interventions that had the highest potential yield in terms of carbon sequestration or avoided emissions. While the addition of more interventions surely would increase the size of the carbon mitigation potential (and associated costs), our study is not intended to be a comprehensive assessment of all possible interventions. We still find substantial impacts even with this subset of land management interventions. Further, our economic assessment does not place a monetary value on the many co-benefits created by NCS other than the air and water quality improvements associated with less nitrogen use in the agricultural sector (e.g., less intensive use of land enhances a myriad of ecosystem services not accounted for here, such as habitat provision, air purification, localized flooding control etc.). Therefore, at a cost of $62–$65 society obtains a sequestered tCO_2_e plus some additional, but unknown, ecosystem service improvement benefit.

NCS interventions can be utilized to mitigate future land carbon emissions driven by climate change impacts on ecosystem growth and disturbance. Physical climate changes due to fossil fuel emissions will continue to impact land-based carbon dynamics regardless of how well individual nations and subnational jurisdictions meet their intended emissions goals. We show that land-based climate mitigation strategies are still an effective means of capturing and storing additional carbon, suggesting they are complementary to aggressive reductions in fossil fuel emissions. Our framework may be used to assess spatially-explicit climate change risk and overall efficacy for both project-based NCS and jurisdictional NCS policies, providing policymakers, government agencies, and landowners with information on the relative impacts and economics of nature-based solutions to climate change.

## Methods

The State of California provides a globally relevant model for evaluating NCS and its stability in the face of increasing climate change-induced disturbances. The state has large carbon sink capacity in both forest^21^ and agricultural lands^36^. It is a biodiversity hotspot ranking among the most geographically and ecologically^35, 37, 38^ diverse regions on the planet^39^. Importantly, the state is already experiencing climate change-driven increases in disturbance^40, 41^, with this trend predicted to continue throughout the twenty-first century^35, 37, 38^. In order to meet its ambitious 2045 carbon neutrality target^34^, California is aggressively developing NCS policies, including a recent executive order from the Governor that directs state agencies to implement strategies to store more carbon in the state’s natural and agricultural lands while achieving a 30% conservation target by 2030^42^. At a national scale, the United States has directed the National Climate Task Force to evaluate how to address climate change related threats via NCS^43^.

**Figure 4:**
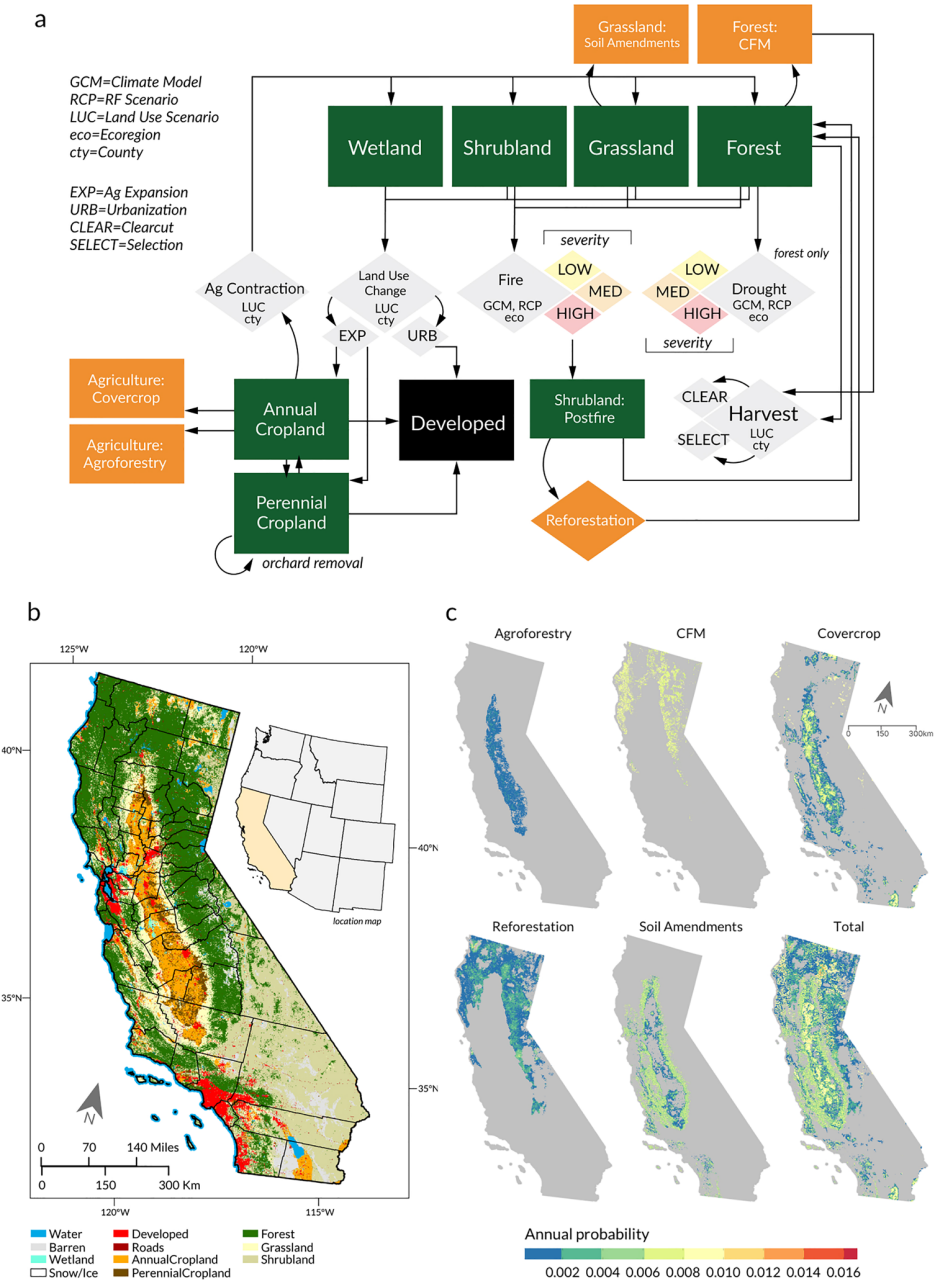
Model diagram. Conceptual diagram of (**a**) state‐and‐transition simulation model, (**b**) map of state classes, (**c**) maps of average annual probability of transition to one of the land management interventions. Green boxes denote ecosystem state classes, gray diamonds indicate land change transition processes, orange boxes represent intervention state classes, and orange diamonds represent intervention land change transition processes.

We used a stochastic state-and-transition simulation model (Land Use and Carbon Scenario Simulator, or LUCAS) with carbon stocks and flows to track spatial changes in land use, disturbance, and their effect on ecosystem carbon stocks and flows^19, 20^. LUCAS estimates land use and climate change effects annually on vegetation productivity, mortality, respiration, and ecosystem carbon balance—including carbon stored in aboveground live biomass, dead organic material, and soils— on California’s natural and agricultural lands at on 1 km^2^ model grid “cells” for the period 2001–2100. We used data on land use and vegetation, climate, wildfire, drought-induced tree mortality, forest harvest, agricultural expansion and contraction, and urbanization from 2001 to 2016 to deterministically drive the model. Starting in 2017, spatial projections of annual wildfire, mortality, and climate from a combination of four global climate models (GCM) and one radiative forcing scenario (RCP) are used to project future disturbance and drive processes such as growth, litter decay, and soil respiration. Figure 4 provides a conceptual diagram of the state-and-transition model. See Sleeter et al.^20^ for complete details on model construction, validation, historical data, and future projections of wildfire, mortality, and climate.

### Climate scenarios

Downscaled CMIP5^44^ climate data from the Localized Construction Analogs (LOCA) dataset were used to represent future climate conditions for the RCP 4.5 radiative forcing scenario^45^. The RCP 8.5 scenario was not included due to computational constraints (each RCP-GCM combination requires at least 800 model runs), in addition to its increasing recognition as an unrealistic future emissions pathway^46^. Climate models chosen represent “hot-dry” (HadGEM2-ES), “hot-wet” (CNRM-CM5), “average” (CanESM2), and “complementary” (MIROC5) climate scenarios, which were the subset of GCMs selected for the California Fourth Climate Change Assessment as models meant to represent a range of possible futures for the state^47, 48^. We included a CO_2_ fertilization effect (CFE) on ecosystem carbon balance. For every 100 ppm increase in atmospheric CO_2_ concentration in each climate future, we increased net primary productivity (NPP) by 5%^20^.

### Baseline land use change

To assess the effects of land management interventions, we created a baseline—or a model simulation with no land management interventions—scenario for each climate future realized through a GCM. We combined each climate future with a “business-as-usual” land use change scenario, which represents the continuation of recent historical rates of land use change. At each annual timestep, the model samples from historical distributions of annual rates of urbanization (1992–2012), agricultural expansion and contraction (1992–2012), and forest harvest (2002–2014) for clearcut and selection harvest types separately. For each baseline scenario we ran at least 50 Monte Carlo realizations to evaluate uncertainty in ecosystem carbon as a function of land use change, wildfire, and drought-induced tree mortality.

We incorporate wildfire disturbance using an exogenous statistical submodel that estimates annual burned area based on effects of climate, vegetation, population density, and fire history^37^. For each GCM, we summarize annual projected burned area by ecoregion and simulate individual wildfire events as described in Sleeter et al.^20^. During the historical period, ecoregion-specific fire severity distributions are calculated based on actual distributions of fire severity from a national database of wildland fire^49^. To account for an expected increase in the proportion of high severity wildfire^50^, we used an annual scalar of 0.82% to create ecoregion-specific increases in high severity wildfire. We based this scalar on a linear model fit to the most recent 20 years of data on fire severity^49^ in California (1997–2016). Forest recovery is not automatic after a high severity wildfire occurs, instead all cells that experience high severity fire are put into a temporary post-fire successional class. Based on recent research from western US forests, these post-fire successional cells are probabilistically allowed to revert to forest with a probability of 0.064 ± 0.027 SD yr^−1^ (75% cumulative recovery over a 20 year period) following the fire. This probability is conservative and is based on the percentage of sites that did not meet a stand recruitment threshold of 50% of pre-fire density^51^. Cells permanently shift to a shrubland class if they do not recover as forest within 20 years.

### Land management interventions

Starting with the baseline land use change model, we added six land management interventions (agroforestry, changes to forest management, cover cropping, reforestation, soil amendments, and land conservation), either in isolation (individual NCS intervention scenarios) or all at once (combined NCS intervention scenario). See *Supplementary Methods* for detailed description of each land management intervention. Other than the changes prescribed by the land management intervention(s) the NCS intervention scenarios are the same as the baseline scenario, allowing us to isolate the effect of the intervention. Each land management intervention began in 2020 and ran through 2100, except for changes to forest management. We ran the intervention model for each of the four future climate scenarios with 50–100 Monte Carlo realizations, and evaluated change in ecosystem carbon between baseline and intervention models within each climate future. We assessed the effect of delaying statewide implementation of NCS interventions using two additional alternative scenarios: a 10-year delay scenario with interventions starting in 2030 and 20-year delay scenario with interventions starting in 2040. Both scenarios used the same underlying baseline model; they simply begin implementation of NCS interventions at a later date. Annual rates of intervention implementation are the same across delayed start scenarios. We limit our analysis of these land management interventions to their impact on biospheric carbon; we do not assess fossil fuel displacement from bioenergy, building material energy use from reduced or increased harvested wood products, or other non-biological carbon flows.

### Economic assessment

We performed an economic impact assessment of each NCS intervention by calculating the direct benefits (societal benefits generated by carbon sequestration) less the direct costs to implement an intervention. We also calculated indirect market costs and benefits—or the changes an NCS intervention has on California’s agriculture, residential development, and forestry markets. We also calculated the relative social cost of nitrogen emissions incurred or avoided as a result of an intervention. For each intervention scenario, including the reference scenario, we estimated the 2020–2050 annual changes in carbon sequestration value, implementation cost, market values, and nitrogen emission costs—discounted at an annual rate of 5%. We then subtracted the net present value of the annual changes in carbon sequestration, implementation cost, market values, and nitrogen emission costs generated by the reference scenario from those generated by an NCS intervention scenario to find the relative economic impacts of the intervention. Additional details can be found in *Supplementary Methods*.

We use a 5% per annum discount rate because the alternative to NCS interventions over the next 30 years are immediate (2020 to 2050) investments in agriculture, housing development, and intensive forestry (recall our economic assessment only extends to 2050). Such near-future land investment decisions are made by private decision-makers who face discount/interest rates in capital markets of at least 5% per annum. Therefore, the cost of NCS interventions also have to be evaluated with, at a minimum, a 5% per annum discount rate. Conversely, the benefits of NCS interventions are public goods that accrue to all members of global society. When a long-term, global perspective on benefits from climate change mitigation is taken, lower discount rates (e.g., 2–3%) may be warranted^52^. In our main analysis we stick to a 5% rate to discount carbon sequestration benefits despite the well-founded arguments for a lower rate for two reasons. First, it is common practice in benefit–cost analysis to use the same rate to discount benefits and costs. Second, the use of a 5% rate is a conservative choice that guards against false positives—namely, NCS inventions that are admissible according to benefit–cost criteria when they should not be. We conduct an additional analysis that discounts benefits with a 2.5% rate (while still discounting costs at a 5% rate) to show the sensitivity of our results to the choice of discount rate (Tables S4 and S5).

### Supplementary Information

Supplementary Information.

## Data Availability

All data needed to evaluate the conclusions in the paper are present in the paper, the Supplementary Materials, or Data Tables and Data Summaries^53^ stored at https://osf.io/dgj4h/. Any use of trade, firm, or product names is for descriptive purposes only and does not imply endorsement by the U.S. Government.
