# Supplementary material for: Natural climate solutions provide robust carbon mitigation capacity under future climate change scenarios
Source: Sci Rep. 2023 Nov 3;13:19008. doi: 10.1038/s41598-023-43118-6 (PMC10624659; doi:10.1038/s41598-023-43118-6)
Supplement: Supplementary file 1 — Supplementary Information. [file 41598_2023_43118_MOESM1_ESM.pdf]

Supplementary Materials for

Natural climate solutions provide robust carbon mitigation capacity under future climate change scenarios

Authors
David C. Marvin, Benjamin M. Sleeter, D. Richard Cameron, Erik Nelson, Andrew J. Plantinga

This PDF file includes:

Supplementary Results
Supplementary Methods
Supplementary References
Figs S1 to S3
Tables S1 to S14

Data Tables and Data Summaries available at https://osf.io/dgj4h

Any use of trade, firm, or product names is for descriptive purposes only and does not imply endorsement by the U.S. Government.

Supplementary Results

Effect of delayed intervention start

Starting NCS interventions in 2030, a 10 year delay, results in an average of 63 MMT less CO_2e stored across all climate futures by 2050 (Fig. S1, Table S6). This is a reduction of 34% potential carbon storage when compared to starting interventions in 2020. CanESM2 saw the largest difference with 67 MMT less CO_2e stored (-37%), followed by HadGEM2-ES with 66 MMT less CO_2e (36%), and CNRM-CM5 and MIROC5 with 59 MMT less CO_2e (-31% and -32%, respectively).

Delaying NCS by 20 years leads to 90 MMT less CO_2e stored by 2050, or a 49% decrease. CNRM-CM5 saw the largest absolute difference with 98 MMT less CO_2e stored (-51%), followed by CanESM2 with 93 MMT less CO_2e (-52%), HadGEM2-ES with 87 MMT less CO_2e (-47%), and MIROC5 with 82 MMT less CO_2e (-44%).

By the end of the century, the average effect of a 10 year delay is 83 MMT less CO_2e, or 11% reduction, while a 20 year delay means 174 MMT less CO_2e, or a 23.0% reduction (Table S6).

Supplementary Methods

Intervention Scenarios

Changes to forest management (CFM). This intervention alters current forest management practices to increase carbon stocks and reduce harvest volumes by increasing rotation age of forest clearcuts and shifting harvest practices away from clearcutting and other even-aged harvest toward selection harvest practices. We "enrolled" 400 km^2/year for 30 years (i.e., until 2050) into a permanent CFM land-use class, meaning any future timber harvest on these lands followed new timber harvest practices defined below**.** The rate was based on the "moderate" rate used to model CFM effects in ref. [5] and represents 75% of the area per year of improved forest management offset projects enrolled in California from 2013-2015. This intervention is meant to emulate a policy whereby landowners commit to permanently change harvest practices in return for an economic incentive (e.g., forest offset program).

We fixed the annual rate of harvest across both CFM and non-CFM lands at 615 km^2/year starting in 2020. Approximately half of this amount is allocated to lands that have been enrolled in the CFM land use class, which is then proportionally allocated to a 70:30 ratio of selection (223 km^2/year) to clearcut (95 km^2/year) harvest. Harvest that does not occur on CFM-enrolled lands is allocated based on the statewide historical 60:40 ratio of clearcut (177 km^2/year) to selection (120 km^2/year) harvest. To simulate extended rotation length, CFM forest cells had the minimum age of clearcut harvest increased to 60 years, up from 40 years in non-CFM lands.

This intervention was restricted to private or tribal forest lands classified as suitable for timber harvest[54] in ecoregions that have a high proportion of forest cover (Sierra Nevada, Northern Basin, Klamath, Eastern Cascades, Coast Range, Central Basin, Cascades). Enrollment of new forest cells in CFM ends in 2050, but lands already enrolled in the CFM regime stayed enrolled until the end of the century. We allowed for reversals out of the CFM class at a rate of 1% per year.

Post-wildfire reforestation. This intervention involves the active replanting of trees in areas that burned under high severity wildfire. This reforestation intervention is meant to automatically shift post-wildfire shrubland cells to a regrowing forest within the 20-year period after the wildfire. We used a rate of 195 km^2 annually based on the ambitious scenario rate used for a similar intervention in ref. [5]. The amount of reforestation annually is allocated among ecoregions based on their proportional forest area: Sierra Nevada (82 km^2/year), Northern Basin (3 km^2/year), Klamath (55 km^2/year), Eastern Cascades (23 km^2/year), Coast Range (21 km^2/year), Central Basin (9 km^2/year), Cascades (2 km^2/year). All cells in the post-fire state classified as forest were eligible for the reforestation intervention, except those that fall within areas identified as being greater than 95 percent unlikely to support the current vegetation type under future climate scenarios[55], as reforesting these areas was assumed to be unproductive. Additionally, cells within protected areas (GAP status 1 or 2) were excluded from reforestation[56].

Cover cropping. This intervention introduces a rotation of non-cash crops—such as winter peas, clover, barley, and rye—on annual agriculture (often planted during winter months) when an

agricultural field would normally lay bare. Prior to cash crop planting the cover crop is plowed under, increasing the amount of organic material incorporated into the soil. Soil carbon is increased through the breakdown of organic material (both roots and above ground components of the plant) after incorporation into the soil. The contribution of the cover crop organic material can substantially increase inputs to the soil carbon pool. All annual agriculture cells were eligible for cover cropping, and we allocated 229 km^2/year to this intervention. Annual agriculture cells that receive this intervention continue to have cover cropping applied each year into the future. This rate was 200 percent of (or 3x) the current rate of cover crop adoption in California as reported by the NRCS[57], a reasonable increase given it's currently low prevalence (<20,000 acres).

On cover crop cells one-third of annual carbon that is normally harvested and removed as straw is instead moved to the litter pool. This increases the amount of soil carbon that cycles through to the soil carbon pool, increasing total soil carbon relative to annual agriculture that does not utilize cover cropping. Since normally the harvested straw does not count toward total ecosystem carbon, this is in effect simulating a scenario where one 4-month season of cover crops are grown and incorporated into the soil, resulting in an increase in soil carbon compared to non-cover crop annual agriculture cells.

Agroforestry. This intervention models the establishment of trees along agricultural field boundaries to act as a windbreak, representing a substantial increase in carbon on landscapes that currently do not hold large quantities of woody above and belowground carbon. This represents the highest carbon sequestration potential of the broader set of agroforestry practices that can occur on fields still primarily used for growing cash crops. We implemented agroforestry practices at a rate of 32 km^2/year, with only annual agriculture cells eligible for agroforestry establishment. The state class was changed from agriculture to forest on cells selected for this intervention. This intervention only occurs in the Central Valley ecoregion—thereby limiting the growth rate and carbon flows to the parameters specific to the Central valley ecoregion. These plantings were assumed to occur at the margins of agricultural fields and other non-productive areas, resulting in no loss to agricultural productivity. We estimated that 7 percent of annual agricultural land area could be planted with windbreaks. This intervention aimed to plant windbreaks on 80 percent of all potential area by 2100.

To calculate the total potential area available for windbreak planting, we assumed an average agricultural field size of 16 hectares, calculated from a field boundary dataset developed by the California Natural Resources Agency [https://data.cnra.ca.gov/dataset/statewide-crop-mapping]. The resulting total linear planting per km^2 of agricultural fields is 75,000 m^2, and assuming a windbreak planting width of 15-meter at field boundaries, there is approximately 7.5 hectares (7.5%) of potential windbreak planting available per km^2. We conservatively rounded this down to 7 percent to arrive at a figure for total potential windbreak planting availability.

Rangeland soil amendments. This intervention involves the addition of organic and mineral soil amendments—materials such as municipal composted biosolids, manure, biochar, pulverized rock, among others—to nonirrigated grassland sites. The increased carbon sequestration from the addition of soil amendments to rangelands results from the stimulation of net primary

productivity due to increased nutrient availability and changes to the soil microenvironment (e.g., water availability, alkalinity, temperature). We based the calculated reductions on ongoing net sequestration of carbon in rangeland soils relative to controls, which are net of any carbon input from the amendments. We applied soil amendments at a rate of 250 km^2/year, only within grassland land cover types in the Central Valley and the Chaparral and Oak Woodland ecoregions, based on an estimate of 50% of suitable land based on municipal compost supply[58]. On cells that received soil amendments, we increased annual NPP using an annual scalar calculated from the DayCent model[59]. Using 7 different locations in California, we used DayCent to estimate NPP through 2100 with and without soil amendments. We calculated the change in NPP relative to no soil amendments applied. The result is an immediate increase in NPP of 15%, peaking at 20% after 25 years, and slowly declining by 2% per decade (Fig. S2).

Land conservation. This intervention is designed to reduce rates of natural land conversion to developed or agricultural land use and reduced rates of agriculture transition to developed land use. We did not implement an annual rate of intervention for land conservation. Instead, following the methods of a recent paper[60], a low population growth scenario was used to deterministically set the annual urban growth rate at the county level. This resulted in an average urbanization reduction of 75 percent over the period 2020-2100 compared to the "business-as-usual" land-use scenario. Similar to ref.[20], to reduce agricultural expansion, an annual conversion rate was sampled from a historical period with relatively low annual agricultural expansion (1993-1996), resulting in a 55 percent reduction in agricultural expansion compared to sampling from the full historical period (1993-2012).

Economic Assessment

We assessed the 2020-2050 economic impact of intervention scenarios. The direct economic benefit of an intervention scenario is the societal value generated by the additional carbon sequestered on the intervention scenario landscape relative to the reference landscape. The direct cost of an intervention scenario is the sum of its implementation costs, such as the expenditures on planting trees following wildfire, establishing windbreaks, cover cropping, etc. In addition, an intervention scenario creates changes in other California markets and these changes have economic effects, referred to as indirect costs. For example, if an intervention scenario means less land in agriculture relative to the reference scenario but crop prices remain constant then the foregone profits from California agriculture are an indirect cost of the intervention scenario. An intervention scenario can also indirectly increase a market's value, as in the case where an intervention scenario landscape includes more managed forest land than the reference scenario but timber prices remain constant. In this research, we estimated the relative impact that the intervention scenarios had on California's agriculture, residential development, and forestry market values. Moreover, we quantified the relative social cost of nitrogen emissions associated with each intervention scenario.

Our estimates account for changes in market values due to changes in the quantities of commodities produced. Our economic model assumes that commodity prices remain fixed at base year levels over time. This generates two sources of error. First, prices change over time partly due to national- to global-level trends that actions in California do not affect. However,

these price changes affect all of our scenario-level economic estimates equally and, as long as we limit ourselves to relative scenario comparisons, this source of error is mitigated in our economic analyses. Second, in some cases, changes in commodity production in California can affect commodity prices, including when commodity production in California represents a disproportionate share of global supply (e.g., California accounts for about 80% of global almond production) or the good in question is sold in a regional market (e.g., developed land). For example, the NCS interventions reduce agricultural acreage relative to the baseline, which would raise relative almond prices. In addition, relative prices for developed land would increase under the NCS interventions, which decrease developed land area. In both cases, indirect costs would be higher with price adjustments, implying that the estimated costs of the NCS interventions are understated.

Not accounting for productivity changes and land manager adaptation caused by continuing climate change is a third source of error in our economic model. Continuing climate change will mean that actual economic productivity inputs such as crop yields and tree growth rates (in fact, any weather-impacted input) will diverge from the values we assume in our assessment (current as of circa 2020). In addition, land managers will adapt to continuing climate change by adopting behaviors (e.g., changing crop choices, changing rotation lengths, etc.) that our economic model does not account for. Indirect costs are likely to go up as climate change decreases agriculture and forestry productivity, implying that the estimated costs of the NCS inventions are understated. However, some of these productivity losses will be mitigated by adaptive behavior, implying that the downward bias in our cost estimates due to unaccounted for climate change could be relatively minor.

Therefore, to summarize, our approach captures the first-order economic impact created by changes in levels of commodity production. However, our approach ignores the second-order economic impacts caused by endogenous price changes, climate change-induced land productivity changes, and climate change-induced land manager adaptation. Nevertheless, these first-order effects dominate the second order effects in aggregate economic impacts. Further, the total economic error generated by ignoring the second-order impacts is attenuated by some conflicting error signs across the suite of second-order impacts. In other words, our scenario-level economic estimates relative to baseline values accurately approximate the magnitude of scenario impacts.

All scenario-level economic values, whether they be a direct benefit or cost of an intervention scenario or the indirect cost of an intervention scenario, were constructed in a similar manner. First, for each intervention scenario, including the reference scenario, we estimated the 2020-2050 annual changes in carbon sequestration value, implementation cost, and market values. Values over time were discounted using a 5% per annum discount rate. Discounting places higher weight on present costs and benefits in order to represent the indirect cost of spending funds today and foregoing investment that would result in higher future wealth. Second, we subtracted the net present value of the annual changes in carbon sequestration, implementation cost, and market values generated by the reference scenario from the respective net present values generated by an intervention scenario to find the scenario’s relative economic impacts (Fig. S3).

Direct economic benefit. The calculation of the societal benefit generated by additional carbon sequestration uses two key inputs. First, we calculated the series of annual net changes in carbon stored on the landscape over the 2020 to 2050 period for each intervention scenario as well as the reference scenario. Due to uncertainty in the biophysical model, we computed the series of mean annual net changes as well as lower and upper bounds on annual net changes for each scenario. Second, we created a series of annual Social Cost of Carbon (SCC) values (2017 USD) for the years 2020 to 2050 and a 5% discount rate[61]. The SCC is an estimate of the present monetary value of a unit of carbon emitted to or absorbed from the atmosphere at a given point in time (SCC measures damage to the global economy when the net change in carbon is negative and measures avoided damage or benefit to the global economy when the net change in carbon is positive). Finally, we multiplied a scenario's series of annual net changes in stored carbon by the annualized SCC value series, discounted each year's monetary product to the year 2020, and then summed the series of discounted values to generate the scenario's direct economic benefit (in 2017 USD). In the results section we report an intervention scenario's direct economic benefit less the reference scenario's direct economic benefit.

Direct implementation costs. The costs of implementing agroforestry, reforestation, and soil amendments projects on the landscape are one-time expenditures that occur in the year the project is undertaken. Conversely, the practice of cover cropping requires annual expenditures that only end once the practice is discontinued. Therefore, total intervention cost under a scenario in a given year includes all the expenditures on that year's new agroforestry, reforestation, and soil amendments projects and the area across the state enrolled in cover cropping in that particular year (per-area cost of each intervention type in 2017 USD are presented in Table S10). Then we discounted the series of intervention costs generated by a scenario each year between 2020 to 2050 to the year 2020 and then summed the series of discounted values to obtain the present value of the scenario's direct economic cost. The intervention costs in the reference scenario are 0. Lower and upper bounds on the direct economic cost estimates for each intervention scenario reflect the potential variation in land area treated with the various interventions. Note that land conservation interventions do not have direct implementation costs, but do have indirect costs associated with foregone profits from agriculture and development, as discussed below.

Value of land in agriculture. Using University of California-Davis enterprise budgets[62,63], we calculated region-level annual net returns to the various crops grown across California (e.g., avocados, grapefruits, lemons, oranges, grapes, hay, tomatoes, etc.). We also estimated region-level annual net returns to pasture and rangelands using data from California land appraisers[64,65] and determined 2016 county-level acreage for each of these crops[66]. Then, using these data, we calculated county-level mean area-weighted per km^2 annual net returns to the agriculture types tracked in the biophysical model - perennial, annual, and grassland. We assumed that the real (inflation-adjusted) annual net return to each agriculture type remains constant during the 2020 to 2050 period.

Next, we multiplied a scenario's series of county-level annual net changes in perennial, annual, and grassland km^2 by the discounted stream of annual net return a km^2 of perennial, annual,

and grassland agriculture is expected to generate in that county between the year of change and 2050, and then summed the series of discounted values across all counties and agricultural types to generate the scenario's estimated impact on 2020 to 2050 economic returns from California's agriculture sector (in 2017 USD). In the results section we report an intervention scenario's relative impact on California's agricultural sector less the reference scenario's relative impact. An intervention scenario entails indirect costs when it generates less agricultural net returns than the reference scenario. Lower and upper bounds on a scenario's relative impact reflect the potential variation in land area that is used for agriculture.

Value of land in development. We calculated the annualized value of a development right on agricultural land in each California county for the years 2007 and 2012 following the approach in ref. [67] using data from the Census of Agriculture on the market value of farmland and farmland acres and estimates of country-level annual net returns to agriculture (discussed above). The average of the 2007 and 2012 country-level values, expressed in 2017 USD, are our estimates of the annualized value of a km^2 of development in each county. We assumed the annualized value of development remained constant during the 2020 to 2050 period.

Next, we multiplied a scenario's series of county-level annual net changes in developed km^2 by the discounted stream of annual net returns a km^2 of development is expected to generate in that county between the year of change and 2050, and then summed the series of discounted values across all counties to generate the scenario's estimated impact on the net returns from California land development from 2020 to 2050 (in 2017 USD). In the results section we report an intervention scenario's relative impact on California's development value less the reference scenario's relative impact on California's development value. An intervention scenario's impact on California 2020-2050 development value is beneficial when it generates more in additional development value than the reference scenario. Lower and upper bounds on a scenario's relative impact on Calirfonia's development value reflect the potential variation in land area that is used for development.

Value of land in managed forestry. First, using the biophysical model, we determined the percentage of forest of each age that underwent a management action of "forest clearcut" or "CFM: clearcut" harvest in each county in each year under each scenario. We use the term "clearcut" in reference to multiple silvicultural practices where even-aged harvest techniques dominate. For example, under the 2020 intervention scenario, 0.44% (or 0.046 km^2) of all forest in Placer County that underwent clearcut harvest in 2020 was 60 years old.

Then, again using the biophysical model's output, we found the area of forest in clearcut management in each county in each year under each scenario. For example, under the 2020 intervention scenario, 779 km^2 of forest in Placer County was under clearcut management in 2020. We then allocated rotation ages across the clearcut managed-forest in a county under a scenario according to the scenario-year-county distribution of rotation ages that experienced a clearcut (see the paragraph above). For example, of the 779 km^2 of forest in clearcut management in Placer County in 2020 under the 2020 intervention scenario, we assumed that 779 x 0.0044 = 3.4 km^2 of it was being managed with a 60 year rotation age. Then, using data on county- and species-specific tree growth rates and timber prices in California for managed

tree types[68,69] data on county-level tree species mix in California as of the mid 2010s[70] and the assumed rotation ages across all clear-cut managed stands in the county, we determined the annualized net return generated by clearcut-managed forest in each county in each year under each scenario. Note that we assume the nonlinear stand growth curves, stumpage prices, and tree species mix does not change over time

Next, for each scenario, we determined each county's annual change in annual net return to clearcut-managed forest between 2020 and 2050 (discounted back to 2020), multiplied each value in a county's series by the discount factor that extends the annual change in annual net returns between the year of change and 2050, and then summed the series of discounted values across all counties. This sum indicates the relative impact a scenario has on 2020-2050 clearcut forestry net returns (in 2017 USD) in California. In the results section we report an intervention scenario's relative impact on California's clearcut forestry net returns less the relative impact under the reference scenario.

We also report each intervention scenario's impact on California's selection forestry 2020-2050 net returns using the same steps as above. In this case, the distribution of rotation ages in each county each year under each scenario is indicated by the ages of forest that underwent a management action of "forest selection" or "CFM: selection." We calculated the annualized net return each piece of land in selection forestry generates by modifying the net return formula to account for the less intensive cutting and additional costs associated with selective forestry relative to clearcut management.

The societal cost of nitrogen fertilizer use. Using University of California-Davis enterprise budgets[62,63], we calculated region-level annual N application rates to the various crops grown across California. Then, using these data along with the data on county-level acreage for each of these crops we collected to model the agriculture sector, we calculated county-level mean area-weighted per km^2 annual N application rates for the agriculture types tracked in the biophysical model - perennial, annual, and grassland.

According to ref. [71] the mean annual social cost of an applied pound of nitrogen (SCN) in Minnesota circa 2010 was $18.68 (2017 USD). This SCN assumes that a pound of applied N creates constant social damages for 20 years at a 5% per annum discount rate. We assumed Minnesota-level SCN values hold in California.

Next, we multiplied a scenario's series of county-level annual net changes in perennial, annual, and grassland km^2 by 1) the estimated county-level N application rates for each agricultural type and 2) the SCN, discounted each county-year's monetary product to the year 2020, and then summed the series of discounted values across all counties and agricultural types to generate the scenario's relative impact on the societal cost of nitrogen fertilizer use from 2020 to 2050 (in 2017 USD). In the results section we report an intervention scenario's relative impact on the societal cost of nitrogen fertilizer use less the relative impact under the reference scenario. An intervention scenario's impact on the societal cost of nitrogen fertilizer use is beneficial when it generates *less* in additional societal damage than the reference scenario. Lower and upper

bounds on a scenario's relative impact on the societal cost of nitrogen fertilizer use reflect the potential variation in land area that is used for agriculture.

The economic value of land use transitions. The transition from one land cover to another, for example, managed forestry to agriculture, agriculture to development, or clearcut forestry to selective forestry, will generate short-term benefits and costs that our economic assessment does not account for. For example, removing forest for development or agricultural use could require extensive stumpage removal and land grading costs. Further, our model assumes changes in land cover and related land uses are instantaneous. However, a change in land cover and related land use can take several years to implement.

Economic Model

In this analysis we present the discounted sum of annual changes from Scenario 's' (a unique GCM-RCP-Intervention combination) less the discounted sum of annual changes from "no intervention" (Fig. S3) where all values after 2020 are discounted to 2020 when characterizing the relative impact of intervention Scenario 's' on California's agriculture market (in our full analyses we use scenario results up to 2050). We use this same method for calculating the carbon sequestration value of a scenario (the direct benefit of a scenario) and every indirect market impact we analyze.

When calculating a scenario's direct implementation cost we assume that every cost in every year is additional. In other words, the height of each bar in the direct implementation cost version of Fig. S3 for Scenario 's' is equal to that year's total implementation cost under Scenario 's'.

The direct economic benefit of an intervention scenario

Annual net change in kilotons of terrestrial-stored carbon

First, we calculated the mean and bounded levels of terrestrial-stored carbon across California each year t under Scenario 's'. Let the state-level mean and bounded values of terrestrial-stored kilotons in year t under Scenario 's' be given by lbC_{ts}, $meanC_{ts}$, and ubC_{ts}, respectively.

The lower bound on stored kilotons in county k in year t under Scenario 's' was calculated using the formula,

$$lbC_{kts} = \text{mean } C_{kts} - ((\text{variance in } C_{kts})^{0.5} \times 1.96) \quad (1)$$

The upper bound on stored kilotons in county k in year t under Scenario 's' was calculated using the formula,

$$ubC_{kts} = \text{mean } C_{kts} + ((\text{variance in } C_{kts})^{0.5} \times 1.96) \quad (2)$$

Next we calculated the lower bound on the annual change in kilotons stored state-wide under Scenario 's' with,

$$lb\Delta C_{ts} = \sum_{k=1}^{K} lbC_{kts} - lbC_{kt-1s} \quad (3)$$

for the years 2020 through 2050. We calculated meanΔC_{ts} and ubΔC_{ts} similarly.

The social cost of carbon in California
In Table S8 we indicate the estimated social cost of a metric ton of CO_2 (SC-CO_2) (2017 USD) in California for the years 2020, 2025, and 2030 under three different discount rates.

Using the multiplier 44/12, we translated California-level SC-CO_2 values into California-level social cost of a metric ton of C (SCC) values (2017 USD) (Table S9). Finally, we calculated a SCC for each year 2020 through 2050 that was not 2020, 2025, or 2030 by assuming a linear trend in SCC between 2020 and 2025 and 2025 and 2030 and that all SCC values after 2030 are equal to 2030 values. Therefore, the SCC values we use for the years 2030 through 2050 are a lower bound on the estimated trajectory of California-level SCC.

The economic value of sequestered carbon
The lower bound on the present value (year 2020) of the economic value created by the net change in terrestrial-stored carbon across California from 2020 to 2050 under Scenario ‘s’ (2017 USD) is given by,

$$lbCV_{s} = 1000 \sum_{t=2020}^{2050} \frac{lb\Delta C_{ts} SCC_{t,5}}{1.05^{t-2020}} \quad (4)$$

where $SCC_{t,5}$ indicates the California SCC in year t assuming a 5% per annum discount rate (we use the 5% per annum discount rate SCC to be consistent with our use of the 5% discount rate throughout this analysis) and the 1000 in the equation converts lbΔC from kilotons of C to Mg of C. The mean and upper bound on the present value (year 2020) of the economic value created by the net change in terrestrial-stored carbon across California from 2020 to 2050 under Scenario ‘s’, meanCV_s and ubCV_s, were calculated similarly.

The direct economic cost of an intervention scenario

The cost of an intervention
The methods we used to estimate the cost of four of the five interventions on the landscape, cover cropping, agroforestry, reforestation, and soil amendments, are described in this section. The fifth intervention, CFM, is accounted for when valuing the economic value of managed forest land.

Three of the interventions modeled here, agroforestry, reforestation, and soil amendments, create a cost that occurs the year the intervention takes place. Specifically, if a piece of land is treated with agroforestry, reforestation, or composing in year t the cost of this intervention is

limited to year t. Conversely, if a piece of land is treated with cover cropping in year t it is treated with cover cropping every year hence (it is an annual treatment upon adoption).

Annual ac^{-1} costs (2017 USD) for the four interventions other than CFM are given in Table S10. We use each intervention's mean ac^{-1} cost in the economic estimates presented later in this paper.

Annual net change in treated area and the resulting economic cost

Let $lbInt_{zts}$, $meanInt_{zts}$, and $ubInt_{zts}$ be the lower bound, mean, and upper bound estimates of the acreage treated with agroforestry, reforestation, or soil amendments (intervention type is indexed by z) in year t under Scenario 's' (Data Table 1). Therefore, the present value (year 2020) of the lower bound on the economic cost (2017 USD) created by intervention z from 2020 to 2050 under Scenario 's' is given by,

$$lbCInt_{zs} = \sum_{t=2020}^{2050} \frac{lbInt_{zts} C_z}{1.05^{t-2020}} \tag{5}$$

where C_z is the estimated one-time ac^{-1} cost of intervention z (see the "Mean" column in Table S10). The present value (year 2020) of the mean and upper bound economic cost created by intervention z across California from 2020 to 2050 under Scenario 's', $meanCInt_{zs}$ and $ubCInt_{zs}$, were calculated similarly.

We did not include cover cropping in the methodology described immediately above because cover cropping is an annual treatment after adoption and therefore requires a different valuation method than the agroforestry, reforestation, and soil amendments interventions. Let $lbInt_{ccts}$, $meanInt_{ccts}$, and $ubInt_{ccts}$ be the lower bound, mean, and upper bound estimates of the acreage treated with cover cropping (z = cc) in year t under Scenario 's'. Therefore, the present value (year 2020) of the lower bound on the economic cost (2017 USD) created by cover cropping from 2020 to 2050 for Scenario 's' is given by,

$$lbCInt_{ccs} = \sum_{t=2020}^{2050} \frac{lbInt_{ccts} C_{cc} \frac{1-DF^{2051-t}}{1-DF}}{1.05^{t-2020}} \tag{6}$$

where C_{cc} is the estimated ac^{-1} cost of cover cropping (see the "Mean" column in Table S10) and, in this case, DF = 1/1.05. The present value (year 2020) of the mean and upper bound economic cost created by cover cropping across California from 2020 to 2050 under Scenario 's', $meanCInt_{ccs}$ and $ubCInt_{ccs}$, were calculated similarly.

Finally, let $lbCInt_s$ be the California-level present value (year 2020) of the lower bound economic cost (2017 USD) created across interventions agroforestry, reforestation, soil amendments, and cover cropping from 2020 to 2050 for Scenario 's' ,

$$lbCInt_{s} = \sum_{z=1}^{4} lbCInt_{zs} \qquad (7)$$

The values for $meanCInt_s$ and $ubCInt_s$ were calculated similarly.

A scenario's impact on California's agriculture market

Annualized net return to agriculture types

Using University of California-Davis enterprise budgets[62,63], we calculated region-level annualized net returns to the various crops grown across California (e.g., avocados, grapefruits, lemons, oranges, grapes, hay, tomatoes, etc.). We also determined 2016 county-level acreage for each of these crops. Then, using these data, we calculated the area-weighted annualized net returns to broader agriculture types i, including Orchards, Vineyards, Annual, Irrigated Pasture, and Dryland Pasture in each county k (we threw out any individual crop net return values that were less than 0 when calculating the county-level area weighted annualized net returns to the broader agriculture types).

To estimate county-level annualized net returns to the agricultural type Rangeland we used the 2016 and 2017 TRENDS in Agricultural Land and Lease Values from the California Chapter of the American Society of Farm Managers and Rural Appraisers[64,65]. We calculated a low and high annualized rangeland net return value for each county k.

If a county was missing an annualized net return value for one of the agriculture types i because that type had no acreage of that crop type in 2016 we used the average of that crop type's annualized net return values from adjacent counties.

Let ANR_{ik} be the annualized net return ac^{-1} to agriculture type i in county k in 2017 USD (Table S11). In this analysis we assume that ANR_{ik} does not change over time.

Annual net change in agriculture area and the resulting economic values

In order to economically value the change in agricultural land use under Scenario 's' we first determined the mean and lower and upper bound of km^2 in each agricultural cover j (Perennial, Grassland, and Annual) in county k in each year t under Scenario 's' (Data Table 2). In this case, j = Perennial is given by the state class "Agriculture: Perennial;" j = Grassland is the combination of the state classes "Grassland: All" and "Grassland: Composted;" and j = Annual is the combination of the state classes "Agriculture: Annual", "Agriculture: Covercrop", and "Agriculture: Agroforestry."

The lower bound on km^2 in agricultural cover j in country k in year t were calculated using the formula,

$$lbj_{kts} = \max [\text{mean of } j_{kts} - ((\text{variance in } j_{kts})^{0.5} \times 1.96), 0] \qquad (8)$$

The upper bound on km^2 in agricultural cover j in country k in year t were calculated using the formula,

ubj_{kts} = mean of j_{kts} + ((variance in of $j_{kts})^{0.5}$ x 1.96) (9)

Next, we crosswalked agricultural type i to agricultural cover j. There is no agricultural type called Perennial. However, there are agricultural types Orchard and Vineyard, which are both perennial covers. Therefore, we assumed that half of j = Perennial area was in the agricultural type i = Orchard and the other half was in the agricultural type i = Vineyard in every k across all t and s. Specifically, let lbP_{kts}, $meanP_{kts}$, and ubP_{kts} indicate the lower bound, mean, and upper bound, respectively, of acreage in j = Perennial in county k in year t under Scenario 's.' Therefore, half of each P_{kts} estimate (i.e., lbP_{kts}, $meanP_{kts}$, and ubP_{kts}) was assigned to i = Orchard, given by O_{kts}, and the other half to i = Vineyard, given by V_{kts}.

Further, there is no agricultural type called Grassland. However, there are the agricultural types Irrigated Pasture, Dry Pasture, Low-Valued Ranglend, and High-Valued Rangeland. Therefore, we assigned a quarter of j = Grassland to each of these four types in every k across all t and s. Specifically, let lbG_{kts}, $meanG_{kts}$, and ubG_{kts} indicate the lower bound, mean, and upper bound, respectively, of acreage in j = Grassland in county k in year t under Scenario 's.' Therefore, a quarter of each G_{kt} estimate (i.,e, lbG_{kts}, $meanG_{kts}$, and ubG_{kts}) was assigned to i = Irrigated Pasture, given by IP_{kts}, a quarter was assigned to i = Dry Pasture, given by DP_{kts}, a quarter was assigned to i = Low-Valued Rangeland, given by LR_{kts}, and a quarter was assigned to High-Valued Rangeland, given by HR_{kts}.

Next, we assigned all of j = Annual (i.,e, lbA_{kts}, $meanA_{kts}$, and ubA_{kts}) to the agricultural type i = Annual.

Finally, we converted all km^2 values to ac values.

Therefore, the present value (year 2020) of the lower bound on the economic value (2017 USD) created by change in agriculture area between years t-1 and t under Scenario 's' across California is given by,

$$lb\Delta ANR_{ts} = 1.05^{-t+2020} \sum_{k=1}^{K} [ANR_{Ok}(lbO_{kts} - lbO_{kt-1s}) + ANR_{Vk}(lbV_{kts} - lbV_{kt-1s}) + ANR_{Ak}(lbA_{kts} - lbA_{kt-1s}) + ANR_{IPk}(lbIP_{kts} - lbIP_{kt-1s}) + ANR_{DPk}(lbDP_{kts} - lbDP_{kt-1s}) + ANR_{LRk}(lbLR_{kts} - lbLR_{kt-1s}) + ANR_{HRk}(lbHR_{kts} - lbHR_{kt-1s})] \quad (10)$$

where t ranges from 2020 to 2050, k indexes counties, and O, V, A, IP, DP, LR, and HR indexes all the agricultural types that we have economically valued. We similarly calculated $mean\Delta ANR_{ts}$ and $ub\Delta ANR_{ts}$.

The present value (year 2020) of the lower bound on the economic value (2017 USD) created by annual change in California agricultural production from 2020 to 2050 into perpetuity under Scenario ‘s’ is given by,

$$lbAV_{s} = (1/1 - DF)\sum_{t=2020}^{2050} lb\Delta ANR_{ts} \tag{11}$$

where, in this case, DF = 1/1.05. We similarly calculated $meanAV_s$ and $ubAV_s$.

The present value (year 2020) of the lower bound on the economic value (2017 USD) created by annual changes in California agricultural production from 2020 to 2050 for that period only under Scenario ‘s’ is,

$$lbARV_{s} = \sum_{t=2020}^{2050} \frac{1-DF^{2051-t}}{1-DF} lb\Delta ANR_{ts} \tag{12}$$

We similarly calculated $meanARV_s$ and $ubARV_s$. In the text we use ARV values, not the AV values, when reporting the impact of a scenario on agricultural sector performance.

A scenario’s impact on California's developed land market

Annualized net return to development

The ac^{-1} value of a development right in county k in year g (either 2007 or 2012) is given by,

$$VDR_{kg} = (MV_{kg} – (NV_{kg}/0.05)) / FA_{kg} \tag{13}$$

where MV_{kg} is the estimated market value of all undeveloped land and its buildings in county k in year g, NV_{kg} is the net cash farm income from all farm operations in county k in year g, 0.05 is the per annum discount rate, and FA_{kg} is the farm acreage in county k in year g. All values are taken from the U.S. Census of Agriculture for 2007 and 2012[72,73].

We used the California CPI to convert each county’s 2007 and 2012 VDR_{kg} from 2007 and 2012 USD measures, respectively, to 2017 USD measures. Next, we took the average of VDR_{k2007} and VDR_{k2012} to obtain an inflation-adjusted VDR_k for county k (Table S12). Finally, we calculated the ac^{-1} annualized value of a development right in county k (2017 USD) with,

$$NDR_k = 0.05 \times VDR_k \tag{14}$$

In this analysis we assume that NDR_k does not change over time (Table S13).

Annual net change in development area and the resulting economic values

Next, we found the bounded and mean acreage in non-transportation related developed cover in county k in year t under Scenario ‘s’ by summing the lower bound, mean, or upper bound on

the area in the state classes of “Developed: Open”, “Developed: Low”, “Developed: Medium”, and “Developed: High” in county k in year t under Scenario ‘s’ (lbD_{kts}, $meanD_{kts}$, and ubD_{kts}, respectively). Data on area in developed land covers can be found in Data Table 2. The agriculture valuation section’s equations (8) and (9) describe the method we used to calculate lower and upper acreage bounds for non-transportation related development in county k in year t under Scenario ‘s’.

The present value (year 2020) of the lower bound on the economic value (2017 USD) created by change in non-transportation related California development between years t-1 and t under Scenario ‘s’ is given by,

$$lb\Delta DNR_{ts} = 1.05^{-t+2020} \sum_{k=1}^{K} NDR_{k}(lbD_{kts} - lbD_{kt-1s}) \quad (15)$$

where t ranges from 2020 to 2050. We similarly calculated $mean\Delta DNR_{ts}$ and $ub\Delta DNR_{ts}$.

The present value (year 2020) of the lower bound on the economic value (2017 USD) created by change in non-transportation related California development from 2020 to 2050 into perpetuity under Scenario ‘s’ is given by,

$$lbDV_{s} = (1/1 - DF) \sum_{t=2020}^{2050} lb\Delta DNR_{ts} \quad (16)$$

where, in this case, DF = 1/1.05. We similarly calculated $meanDV_s$ and $ubDV_s$.

The present value (year 2020) of the lower bound on of the economic value (2017 USD) created by changes in non-transportation related California development from 2020 to 2050 for that period only under Scenario ‘s’ is given by,

$$lbDRV_{s} = \sum_{t=2020}^{2050} \frac{1-DF^{2051-t}}{1-DF} lb\Delta DNR_{ts} \quad (17)$$

We similarly calculated $meanDRV_s$ and $ubDRV_s$. In the text we use DRV values, not DV values, when reporting the impact of a scenario on the net returns to development.

A scenario’s impact on California's clearcut forestry market

The net present value of a single clearcut

We used a derivation of the classic Faustmann formula (Faustmann 1849)[57] to calculate the ac^{-1} net present value (year 2020) of the economic value created by one clearcut of species group j in forest type f of age T in county k (assuming the stand is re-established after the clearcut at time T).

$$NR(T)_{jfk} = \frac{A_{jfk} f(T)_{jfk} [p_{jfk} - c_k]}{(1+\delta)^{(T-1)}} - 1[NE]_{jfk} A_{jfk} f(T)_{jfk} c_k \quad (18)$$

where A_{jfk} is the number of trees ac^{-1} in a managed stand of j,f in county k; $f(T)_{jfk}$ is the average volume (in ft^{-3}) of a T year-old j,f tree in county k; p_{jfk} is the stumpage price of of j,f in county k (2010 USD ft^{-3}); c_k is the cost of establishing a forest in county k (2010 USD ft^{-3}); δ is the per annum discount rate; and **1**$[NE]_{jfk}$ = 1 if stand j,f in county k has not been established yet at T = 0 and equals 0 otherwise.

There are 202 unique j,f,k combinations in California with 13 species groups (j), 26 forest types (f), and 33 counties (k).

The net present value of an infinite series of clearcuts

The ac^{-1} net present value (year 2020) created by an infinite series of clearcuts (2010 USD), each of rotation age T, is given by,

$$\pi(T)_{jfk} = \frac{Q(T)_{jfk} [p_{jfk} - c_k]}{(1+\delta)^{(T-1)}} \left(1 + \frac{1}{(1+\delta)^{2(T-1)}} + \frac{1}{(1+\delta)^{3(T-1)}} + \ldots\right) - 1[NE]_{jfk} Q(T)_{jfk} c_k \quad (19)$$

where $Q(T)_{jfk}$ equals equation (18)'s term $A_{jfk} f(T)_{jfk}$. Equation (19) simplifies to,

$$\pi(T)_{jfk} = \frac{Q(T)_{jfk} [p_{jfk} - c_k]}{(1+\delta)^{(T-1)} - 1} - 1[NE]_{jfk} Q(T)_{jfk} c_k \quad (20)$$

A net present value-maximizing stand owner that clearcuts an infinite series of rotations will use the rotation length T that maximizes the value of equation (20). Let T^*_{jfk} indicate the specific clear cut rotation length that maximizes $\pi(T)_{jfk}$.

The annualized net present value of a managed infinite series of clearcuts

The annualized ac^{-1} net present value (year 2020) of an infinite series of clearcut rotations is given by $\frac{\delta}{1+\delta}\pi(T)_{jfk}$. If we assume the manager of the stand chooses the profit-maximizing rotation length then the annualized net present value of an infinite series of rotations is given by $\frac{\delta}{1+\delta}\pi(T^*_{jfk})_{jfk}$.

Data to calculate $\frac{\delta}{1+\delta}\pi(T)_{jfk}$ for each unique j,f,k,T combination with and without establishment costs are in Data Table 3 (inflated from 2010 USD to 2017 USD to be consistent with the rest of our analysis). Using this data also we found the net present value-maximizing rotation length T for each j,f,k combination (i.e., T^*_{jfk}) with and without T = 0 establishment costs.

Next, using historic data on the share of j,f stands in each county k (Data Table 4), we calculated j,f-weighted $\frac{\delta}{1+\delta}\pi(T)_k$ for each k,T combination with and without T = 0 establishment costs (Data Tables 5 and 6). Using this data we also found the annualized net present value-maximizing clearcut rotation length T for each county k (i.e., T_k^*) with and without T = 0 establishment costs.

Therefore, the annualized net present value (in year 2020) ac^{-1} of clearcut forest in county k is given by $\frac{\delta}{1+\delta}\pi(T)_k$ or by $\frac{\delta}{1+\delta}\pi(T_k^*)_k$ if we assume the clearcut is optimally managed (both now measured in 2017 USD).

Annual net change in clearcut area and the resulting economic values
First, we summed the area of management actions “Management: Forest Clearcut" and “Management: CFM Clearcut" from the model output Data Table 9 to find the km^2 of forest age T in county k that was clearcut in year t under Scenario ‘s’, given by $CA(T)_{kts}$. Further, let,

$$CA_{kts} = \sum_{T=0}^{200} CA(T)_{kts} \tag{21}$$

indicate the km^2 of clearcut forest in county k in year t under Scenario ‘s’ (we assume that no mamaned tree stands are older than 200 years).

However, this information only gives us data on the amount of forest removed by clearcut in county k in year t (and its age). With this limited information we can only value the flow of timber from k via clearcut in year t under Scenario ‘s’. Instead we want to know the value of all land managed for clearcut forestry in k in year t under Scenario ‘s’. To do this we first have to determine the fraction of clearcut area that is of age T in year t in county k under Scenario ‘s’.

$$FCA(T)_{kts} = CA(T)_{kts}/CA_{kts} \tag{22}$$

Next, we use data on km^2 that is being managed for clearcut forestry in year t in county k under Scenario ‘s’. Let this be represented by MCA_{kts} (km^2; Data Table 10). We assume that the age distribution of MCA_{kts} is the same as the age distribution of $CA(T)_{kts}$. Therefore,

$$MCA(T)_{kts} = MCA_{kts}FCA(T)_{kts} \tag{23}$$

indicates the km^2 of clearcut managed forest that is of age T in year t in county k under Scenario ‘s’.

Next, we calculated the annualized net present value (year 2020) of land managed for clearcut forestry in county k in year t under Scenario ‘s’ 2017 (USD) with,

$$AVCCNE_{kts} = 247.105 \sum_{T=1}^{200} MCA(T)_{kts} AVCCNE(T)_{k} \quad (24)$$

where AVCCNE(T)$_k$ is equal to $\frac{\delta}{1+\delta}\pi(T)_k$ assuming no T = 0 establishment costs (i.e., **1**[NE]$_{jfk}$ = 0) and the constant 247.105 converts km^2 of MCA(T)$_{kts}$ to ac of MCA(T)$_{kts}$ (Data Table 11).

As with all other economic valuations in this research, we are ultimately interested in the annual change in the value of land managed for clear cut forestry under Scenario 's'. Therefore, let the present value (year 2020) of the economic value (2017 USD) created by the change in clearcut management between years t -1 and t in county k under Scenario 's' be given by,

$$\Delta AVCCNE_{kts} = 1.05^{-t+2020}(AVCCNE_{kts} - AVCCNE_{kt-1s}) \quad (25)$$

Therefore, the mean present value (year 2020) of the economic value (2017 USD) created by changes in clearcut forestry from 2020 to 2050 into perpetuity across the state of California under Scenario 's' is,

$$meanCCV_{s} = 1/(1 - DF) \sum_{t=2020}^{2050} \sum_{k} \Delta AVCCNE_{kts} \quad (26)$$

where, in this case, DF = 1/1.05. We also calculated lbCCV$_s$ and ubCCV$_s$ by using the 5th and 95th percentile values of CA(T)$_{kts}$.

The mean present value (year 2020) of the economic value (2017 USD) created by changes in clearcut forestry from 2020 to 2050 for that period only under Scenario 's' is given by,

$$meanCCRV_{s} = \sum_{t=2020}^{2050} \frac{1-DF^{2051-t}}{1-DF} \sum_{k} \Delta AVCCNE_{kts} \quad (27)$$

We similarly calculated lbCCRV$_s$ and ubCCRV$_s$ by using the 5th and 95th percentile values of CA(T)$_{kts}$. In the text we use CCRV values, not the CCV values, when reporting the impact of a scenario on the net returns to clearcut forestry.

A scenario's impact on California's select forestry market

1. clearcuts half of the stand's area; and
2. incurs additional harvest cost relative to clearcut forestry regimes because the selective forestry manager cannot take full advantage of economies of scale in timber harvesting that clearcutters do.

Knoke (2012)[74] estimates the cost of managing selective systems versus clear-cut systems to be an additional 2.5 to 5 euros m^{-3} of timber. We will use the parameter d to represent the additional cost to selective forestry.

To add the cost parameter d to our traditional forestry model (equations (18) - (20)) we first need to convert d from being measured in euros m^{-3} of timber (2012 euros) to being measured in dollars ft^{-3} of timber (2012 USD). There are 0.093 m^3 ft^{-3}. Further, a dollar was worth 0.809 euros in 2012. Therefore, in 2012, 2.5 to 5 euros m^{-3} of timber was equivalent to 0.29 to 0.57 dollars ft^{-3} of timber. Using the California CPI, this range is 0.27 to 0.55 dollars ft^{-3} of timber in 2010 USD (recall equations (18) - (20) gives values in 2010 USD). We set the parameter d equal to the average of the 2010 USD range, 0.41 dollars ft^{-3}.

The net present value of one rotation of a selectively managed forest

Therefore, assuming a selective forester only clearcuts half of their managed forest stand and incurs additional harvest cost of d, the ac^{-1} net present value (year 2020) of one rotation of a selectively managed forest of species group j in forest type f in county k with rotation length of T is (assuming the portions of the clearcut stand stand is re-established at time T),

$$SNR(T)_{jfk} = 0.5\frac{A_{jfk}f(T)_{jfk}\left[p_{jfk}-0.41-c_k\right]}{(1+\delta)^{(T-1)}} - 1[NE]_{jfk}A_{jfk}f(T)_{jfk}c_k \quad (28)$$

where all equation (28) variables are the same as those found in equation (18). In equation (28) we assume that the selective forest is initially established on land that was previously entirely clearcut.

The net present value of an infinite series of selective forestry cuts

The ac^{-1} present net value (year 2020) of the economic value (2010 USD) created by an infinite series of selective forest cuts, each of rotation age T, is given by,

$$\theta(T)_{jfk} = 0.5\frac{Q(T)_{jfk}\left[p_{jfk}-0.41-c_k\right]}{(1+\delta)^{(T-1)}-1} - 1[NE]_{jfk}Q(T)_{jfk}c_k \quad (29)$$

where all equation (29) variables are the same as those found in equation (20). Data to calculate $\theta(T)_{jfk}$ are contained in Data Table 3.

The annualized net present value of an infinite series of selective forestry cuts

Using this data we calculated the ac^{-1} selective forestry annualized profit (i.e., $\frac{\delta}{1+\delta}\theta(T)_{jfk}$) for each unique j,f,k,T combination with and without T = 0 establishment cost (inflated to 2017 USD to be consistent with the rest of our analysis).

Further, using historic data on the share of j,f by county k, we also calculated j,f-weighted $\frac{\delta}{1+\delta}\theta(T)_k$ for each T with and without establishment costs (2017 USD; Data Tables 7 and 8). Using this data we also found the net present value-maximizing selective forestry rotation length T for each county k (i.e., T^*_k) with and without T = 0 establishment costs.

Therefore, the ac^{-1} annualized net present value (in year 2020) of selectively managed forest in county k is given by $\frac{\delta}{1+\delta}\theta(T)_k$ and by $\frac{\delta}{1+\delta}\theta(T^*_k)_k$ (both in 2017 USD) if we assume the selectively harvested forest is optimally managed.

Annual net change in select forest area and the resulting economic values
First, we summed the area of management actions "Management: Forest Selection" and "Management: CFM Selection" from the model output Data Table 9 to find the km^2 of forest age T in county k that was selectively cut in year t under Scenario 's', given by $SA(T)_{kts}$. Further, let,

$$SA_{kts} = \sum_{T=0}^{200} SA(T)_{kts} \tag{30}$$

indicate the total km^2 of selective forestry in county k in year t under Scenario 's'.

However, this information only gives us data on the amount of forest removed by selective management in county k in year t (and the age of the removed trees). With this limited information we can only value the flow of wood coming from county k via selective forestry in year t under Scenario 's'. Instead we want to know the value of all land managed for selective forestry in k in year t under Scenario 's'.

To do this we first have to determine the fraction of selective forestry area that is of age T in year t in county k under Scenario 's'.

$$FSA(T)_{kts} = SA(T)_{kts}/SA_{kts} \tag{31}$$

Next, we use data on the km^2 that is being managed for selective cut forestry in year t in county k under Scenario 's'. Let this be represented by MSA_{kts} (Data Table 10). We assume that the age distribution of MSA_{kts} is the same as the age distribution of $SA(T)_{kts}$. Therefore,

$$MSA(T)_{kts} = MSA_{kts} FSA(T)_{kts} \tag{32}$$

indicates the km^2 of selective forestry managed forest that is of age T in year t in county k under Scenario 's'.

Next, we calculated the annualized value of land managed for selective forestry (2017USD) in county k in year t under Scenario 's' with,

$$AVSNE_{kts} = 247.105 \sum_{T=1}^{200} MSA(T)_{kts} AVSNE(T)_k \tag{33}$$

where AVSNE(T)$_k$ is equal to $\frac{\delta}{1+\delta}\theta(T)_k$ assuming no T = 0 establishment costs (i.e., **1**[NE]$_{jfk}$ = 0; Data Table 12) and 247.105 converts km^2 of MSA(T)$_{kts}$ to ac of MSA(T)$_{kts}$.

For all other economic valuations in this analysis we find the annual change in production value under Scenario ‘s’. Therefore, let the present value (year 2020) of the economic value (2017 USD) created by the change in selective forest area between years t -1 and t in county k under Scenario ‘s’ be given by,

$$\Delta AVSNE_{kts} = 1.05^{-t+2020}(AVSNE_{kts} - AVSNE_{kt-1s}) \tag{34}$$

Therefore, the mean present value (year 2020) of the economic value (2017 USD) created by changes in selective forestry from 2020 to 2050 into perpetuity across the state of California under Scenario ‘s’ is,

$$meanSV_s = (1/(1 - DF)) \sum_{t=2020}^{2050} \sum_k \Delta AVSNE_{kts} \tag{35}$$

We also calculated lbSV$_s$ and ubSV$_s$ by using the 5th and 95th percentile values of SA(T)$_{kts}$.

The mean present value (year 2020) of the economic value (2017 USD) created by changes in selective forestry from 2020 to 2050 for that period only under Scenario ‘s’ is given by,

$$meanSRV_s = \sum_{t=2020}^{2050} \frac{1-DF^{2051-t}}{1-DF} \sum_k \Delta AVCCNE_{kts} \tag{36}$$

We similarly calculated lbCCRV$_s$ and ubCCRV$_s$ by using the 5th and 95th percentile values of SA(T)$_{kts}$. In the text we use SRV values, not SV values, when reporting the impact of a scenario on the net returns to selective forestry.

The social cost of nitrogen fertilizer use

Fertilizer application rates

Using University of California-Davis enterprise budgets[62,63], we found the average amount of nitrogen (N) fertilizer applied annually to an acre of crop r (e.g., avocados, grapefruits, lemons) across the different regions of California. We also determined 2016 county-level acreage for each crop r.[54] Then, using region-level N applications rates for each r and data on 2016 acreage of r in each county, we calculated the area-weighted amount of N applied annually to the broader agriculture types i we model in this research, including Orchards, Vineyards, Annual, Irrigated Pasture, Dryland Pasture, Low Value Rangeland, and High Value Rangeland, in each county k.

Let N_{ik} be the average annual pounds of N applied to an acre of agriculture type i in county k. In this analysis we assume that N_{ik} does not change over time (Table S14).

If a county k was missing an annual N application rate for agriculture type i because k did not include that crop type in 2016 we used the average of i's annual application rate from adjacent counties for N_{ik}.

The social cost of nitrogen

According to Keeler et al. (2016)[59] the mean annual social cost of an applied kg of nitrogen in Minnesota circa 2010 was $2.62, with a range from $0.44 to $10.79 (2010 USD). Assuming a kg of applied N creates constant social damages for 20 years, the mean present value of damage caused by a kg of N in Minnesota assuming a 5% per annum discount rate is,

$$SCN = \sum_{f=0}^{19} \frac{2.62}{1.05^{f}} = 34.28 \quad (37)$$

Furthermore, minimum and maximum SCN are $5.76 and $141.19, respectively. After converting these SCN estimates to $ lb^{-1} of applied N and 2017 USD using the California CPI series, mean SCN is $18.68 lb^{-1}, minimum SCN is $3.14 lb^{-1}; and maximum SCN is $76.91 lb^{-1}. We assumed Minnesota-level SCN values hold in California.

Annual net change in applied N and the resulting economic cost

The present value (year 2020) of the lower bound on the economic value created by change in N application between years t-1 and t under Scenario 's' is given by,

$$\begin{aligned} lb\Delta NC_{ts} = 1.05^{-t+2020} SCN \sum_{k=1}^{K} [&N_{Ok}(lbO_{kts} - lbO_{kt-1s}) + \\ &N_{Vk}(lbV_{kts} - lbV_{kt-1s}) + \\ &N_{Ak}(lbA_{kts} - lbA_{kt-1s}) + \\ &R_{IPk}(lbIP_{kts} - lbIP_{kt-1s}) + \\ &N_{DPk}(lbDP_{kts} - lbDP_{kt-1s}) + \\ &N_{Rk}(lbLR_{kts} - lbLR_{kt-1s}) + \\ &(lbHR_{kts} - lbHR_{kt-1s})] \quad (38) \end{aligned}$$

where lbO_{kts}, lbV_{kts} , lbA_{kts}, $lbIP_{kts}$, $lbDP_{kts}$, $lbLR_{kts}$ and $lbHR_{kts}$ are the lower bounds on area in agricultural types Orchard, Vineyard, Annual, Irrigated pasture, Dry Pasture, Low Value Rangeland, and High Value Rangeland in county k in year t under scarions s. These same acreage estimates were used when we found the lower bound on the economic value of change in agricultural land use between years t-1 and t under Scenario 's'.

We similarly calculated $mean\Delta NC_{ts}$ and $ub\Delta NC_{ts}$ using the mean and upper bound estimates of area in agricultural types Orchard, Vineyard, Annual, Irrigated pasture, Dry Pasture, Low Value Rangeland, and High Value Rangeland in county k in year t under scarions s.

The present value (as of 2020) of the lower bound on the social cost imposed by annual change in N application from 2020 to 2050 for that period only across California under Scenario 's' is,

$$lbNC_{s} = \sum_{t=2020}^{2050} lb\Delta NC_{ts} \quad (39)$$

We similarly calculated $meanNC_s$ and $ubNC_s$.

Supplementary References

54. CALFIRE. Forest Resources Assessment Program, Timber suitability GIS data. (2009).
55. Thorne, J. H. *et al.* The impact of climate change uncertainty on California's vegetation and adaptation management. *Ecosphere* vol. 8 e02021 Preprint at https://doi.org/10.1002/ecs2.2021 (2017).
56. USGS. Protected Areas Database v 1.4. (2016).
57. NRCS. Natural Resources Conservation Service Practices database. (2018).
58. DeLonge, M. S., Ryals, R. & Silver, W. L. A Lifecycle Model to Evaluate Carbon Sequestration Potential and Greenhouse Gas Dynamics of Managed Grasslands. *Ecosystems* vol. 16 962–979 Preprint at https://doi.org/10.1007/s10021-013-9660-5 (2013).
59. Ryals, R., Hartman, M. D., Parton, W. J., DeLonge, M. S. & Silver, W. L. Long-term climate change mitigation potential with organic matter management on grasslands. *Ecol. Appl.* **25**, 531–545 (2015).
60. Sleeter, B. M., Wilson, T. S., Sharygin, E. & Sherba, J. T. Future scenarios of land change based on empirical data and demographic trends. *Earths Future* **5**, 1068–1083 (2017).
61. California Air Resources Board. *California's 2017 Climate Change Scoping Plan. The strategy for achieving California's 2030 greenhouse gas target*. (2017).
62. Murdoch, J. & Goodrich, B. Archived Cost and Return Studies. *UC Davis Agricultural and Resource Economics* https://coststudies.ucdavis.edu/en/archived/.
63. Murdoch, J. & Goodrich, B. Current Cost and Return Studies. *UC Davis Agricultural and Resource Economics* https://coststudies.ucdavis.edu/en/current/.
64. California Chapter of the American Society of Farm Managers and Rural Appraisers, Inc. 2017 Trends in Agricultural Land & Lease Values. https://www.calasfmra.com/trends.php (2017).
65. California Chapter of the American Society of Farm Managers and Rural Appraisers, Inc.

2016 Trends in Agricultural Land & Lease Values. https://www.calasfmra.com/trends.php. (2016).

66. USDA. National Agricultural Statistics Service - Publications - 2018 Agricultural Statistics Annual. https://www.nass.usda.gov/Publications/Ag_Statistics/2018/index.php (2018).
67. Plantinga, A. J. & Miller, D. J. Agricultural Land Values and the Value of Rights to Future Land Development. *Land Econ.* **77**, 56–67 (2001).
68. Mihiar, C. An Econometric Analysis of the Impact of Climate Change on Forest Land Value and Broad Land-use Change. (2018).
69. Faustmann, M. Calculation of the value which forest land and immature stands possess for forestry. *Allgemeine Fotst-und Jagd-Zeitung* **25**, 441–455 (1849).
70. CALFIRE. Multi-source vegetation data (FVEG15). (2015).
71. Keeler, B. L. *et al.* The social costs of nitrogen. *Sci Adv* **2**, e1600219 (2016).
72. US Department of Agriculture. US Census of Agriculture 2007. *Census of Agriculture Historical Archive* https://agcensus.library.cornell.edu/census_year/2007-census/.
73. US Department of Agriculture. US Census of Agriculture 2012. *Census of Agriculture Historical Archive* https://agcensus.library.cornell.edu/census_year/2012-census/.
74. Knoke, T. The Economics of Continuous Cover Forestry. *Continuous Cover Forestry* 167–193 Preprint at https://doi.org/10.1007/978-94-007-2202-6_5 (2012).
75. USDA Natural Resource Conservation Service. *Conservation Stewardship Program Practice Scenarios Cost Estimates*. (2017).
76. California Department of Food and Agriculture. 2020 Healthy Soils Program Incentives Program. Preprint at https://www.cdfa.ca.gov/oefi/healthysoils/incentivesprogram.html (2020).

SI Figures

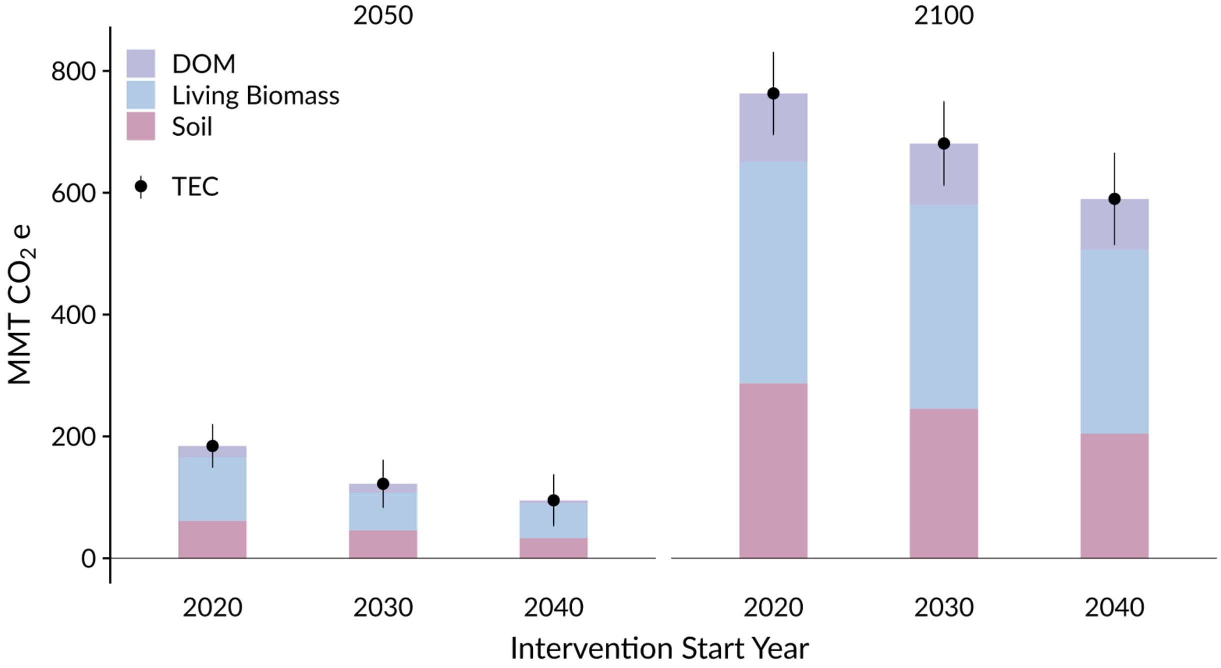

Fig. S1.
Effect of delayed implementation of NCS interventions. Cumulative change in carbon stocks across all climate futures and NCS interventions relative to no interventions with shading showing the 95% confidence interval for each intervention start year. Baseline change is shown for all intervention scenarios starting in 2020 (a). Cumulative change in carbon stock by mid- and end-of-century for each major carbon pool for each intervention start year (b). DOM is dead organic material. TEC is total ecosystem carbon.

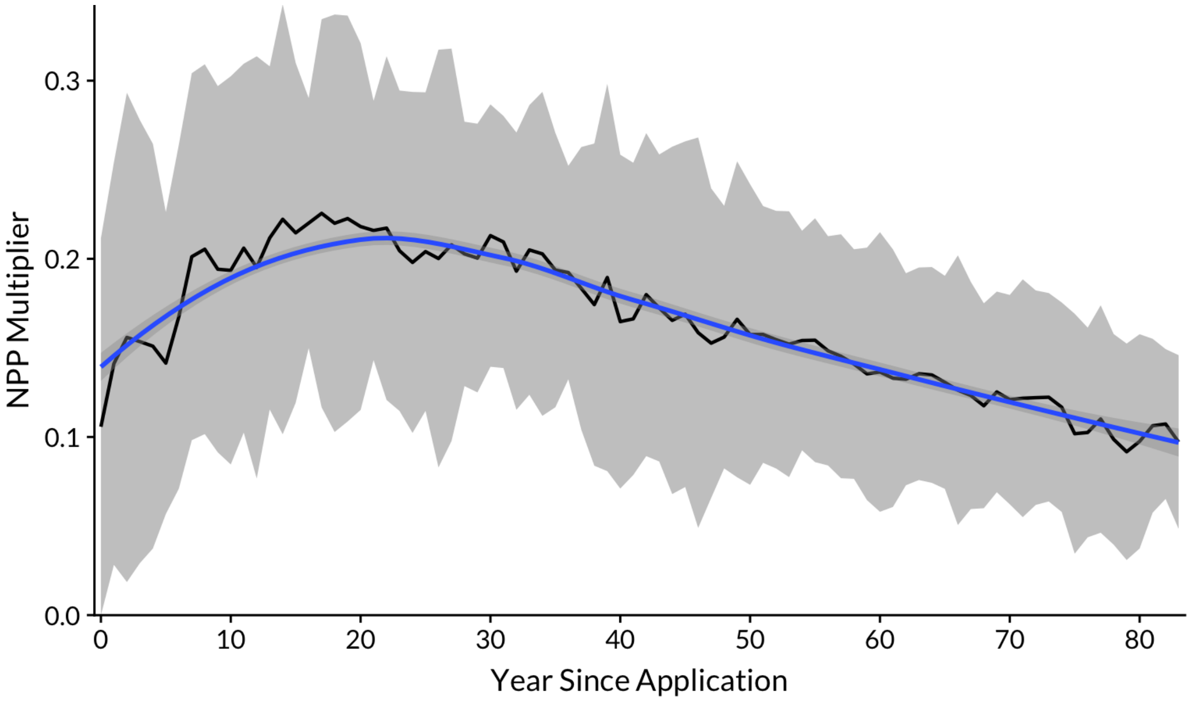

Fig. S2.
Response of grassland net primary productivity (NPP) after rangeland amendments are applied. Black line with gray shading shows the mean +/- 1 SD of the NPP response from DayCent model output. Blue line is a best fit to highlight the temporal response curve after amendment application.

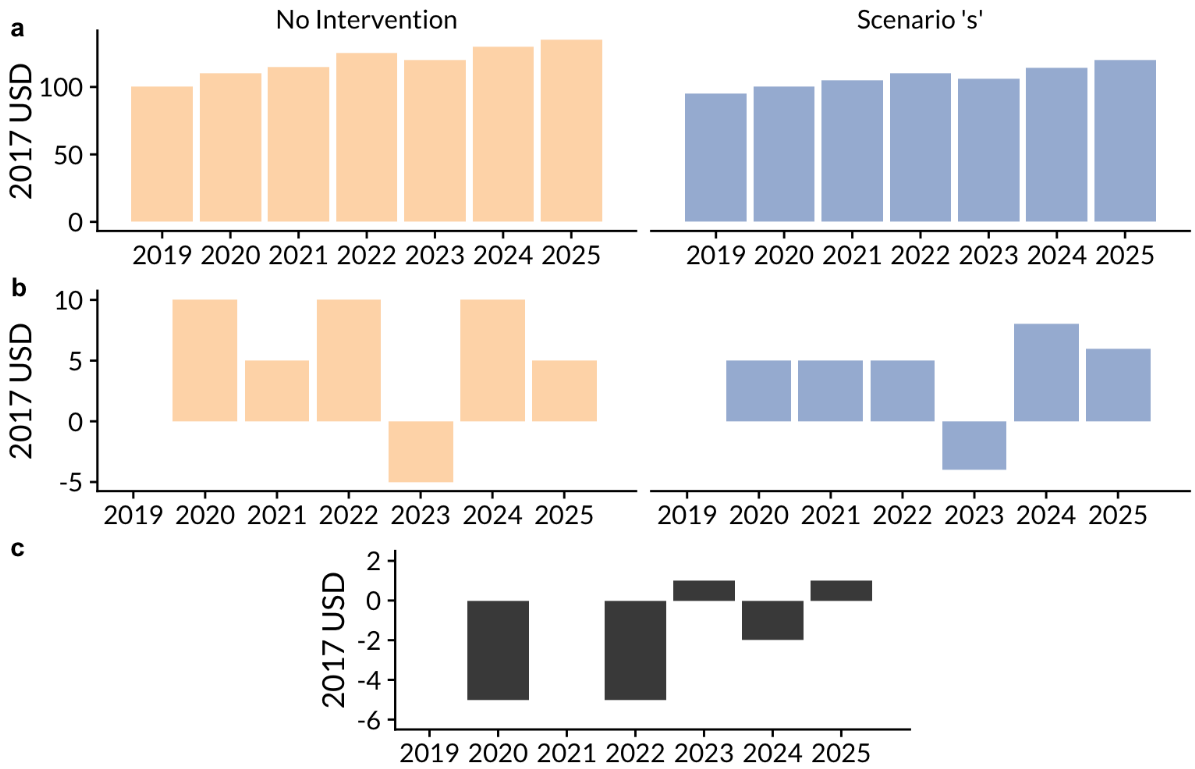

Fig. S3.
These figures illustrate our method for measuring the economic impact of a scenario. (a) Annual net returns (in billion USD) to to a fictitious economic sector in California from 2019 to 2025 under the no intervention scenario and intervention scenario ‘s’, (b) the annual change in annual net returns in the fictitious sector under both scenarios, and (c) the difference in annual change in annual net returns between the scenarios.

SI Tables

Intervention		Climate Future: CanESM2	CNRM-CM5	HadGEM2-ES	MIROC5
		**2050**			
**Forest**		**72 (67, 77)**	**55 (50, 59)**	**68 (66, 69)**	**75 (72, 78)**
	CFM	60 (56, 63)	58 (55, 62)	63 (63, 63)	65 (64, 66)
	Reforestation	13 (11, 14)	-4 (-5, -3)	5 (4, 6)	10 (8, 12)
**Agriculture**		**25 (21, 29)**	**-3 (-5, 0)**	**31 (26, 35)**	**56 (52, 59)**
	Agroforestry	10 (9, 11)	-1 (-1, 0)	0 (-2, 1)	15 (14, 16)
	Cover Crop	7 (6, 8)	4 (2, 5)	13 (11, 14)	31 (30, 32)
	Rangeland Amendments	8 (6, 9)	-6 (-6, -5)	18 (17, 20)	9 (8, 11)
**Conservation**		**84 (83, 86)**	**81 (80, 81)**	**81 (79, 83)**	**92 (91, 92)**
**Intervention**		**2100**			
**Forest**		**303 (301, 305)**	**287 (276, 298)**	**318 (316, 320)**	**309 (305, 314)**
	CFM	128 (126, 130)	135 (130, 140)	146 (145, 147)	141 (139, 143)
	Reforestation	175 (174, 175)	152 (146, 157)	172 (172, 173)	168 (166, 171)
**Agriculture**		**169 (165, 174)**	**176 (175, 177)**	**227 (224, 230)**	**165 (156, 174)**
	Agroforestry	98 (95, 101)	101 (101, 101)	100 (98, 101)	88 (84, 92)
	Cover Crop	56 (55, 57)	83 (82, 83)	92 (91, 93)	70 (68, 71)
	Rangeland Amendments	15 (14, 16)	-7 (-8, -7)	35 (35, 36)	7 (3, 11)
**Conservation**		**268 (267, 269)**	**283 (283, 284)**	**263 (263, 264)**	**252 (246, 259)**

Table S1.
Cumulative emissions reduction potential from individual NCS intervention scenarios. Mean cumulative emissions, in MMT CO_2e, with lower and upper bounds in parenthesis for each climate future and individual intervention. Intervention categories in bold are the sum of individual interventions below the category title. Totals across all individual interventions within a climate future may not exactly match the “no intervention delay” scenario Table S2 because these are separate model runs with the individual interventions run in isolation.

	Climate Future			
	CanESM2	CNRM-CM5	HadGEM2-ES	MIROC5
	**no intervention delay**			
**Direct Cost**				
Total	3926	3794	3855	3765
Annualized	255	247	251	245
**Indirect Cost**				
Agriculture	3492	3755	3831	4574
SCN	*-316*	*-331*	*-321*	*-384*
Developed	259	385	490	450
Forest Harvest	44	64	15	48
Clearcut	*6*	*83*	*-4*	*53*
Selection	*38*	*-19*	*18*	*-4*
Total	3795	4204	4335	5072
Annualized	247	273	282	330
**Direct + indirect Cost**				
Total	7721	7997	8189	8836
Annualized	502	520	533	575
**Carbon Benefit**				
Total	-1435	-1278	-2303	-1451
Annualized	-93	-83	-150	-94
	**10-year intervention delay**			
**Direct Cost**				
Total	1359	1361	1295	1402
Annualized	109	109	104	113
**Indirect Cost**				
Agriculture	719	1633	1261	1648
SCN	*-130*	*-182*	*-188*	*-188*
Developed	239	444	463	525
Forest Harvest	-79	-96	-56	-55
Clearcut	*-38*	*-48*	*-24*	*-30*
Selection	*-41*	*-48*	*-33*	*-25*
Total	878	1981	1668	2118
Annualized	70	159	134	170
**Direct + Indirect Cost**				
Total	2238	3343	2962	3520
Annualized	180	268	238	282
**Carbon Benefit**				
Total	-2836	-2639	-1906	-3105
Annualized	-228	-212	-153	-249
	**20-year intervention delay**			
**Direct Cost**				
Total	261	288	279	273
Annualized	34	37	36	35
**Indirect Cost**				

Agriculture	198	163	170	189
SCN	*-86*	*-90*	*-76*	*-78*
Developed	235	501	430	454
Forest Harvest	-28	-25	-34	-18
Clearcut	*-14*	*-8*	*-15*	*-9*
Selection	*-15*	*-17*	*-20*	*-9*
Total	405	639	567	625
Annualized	52	83	73	81
**Direct + Indirect Cost**				
Total	666	927	846	898
Annualized	86	120	109	116
**Carbon Benefit**				
Total	-2421	-2902	-2970	-3481
Annualized	-313	-376	-385	-451

Table S2.

Estimated minimum indirect costs, direct costs, and carbon benefits of NCS interventions. NCS interventions relative to the no intervention scenario over the period 2020-2050, for no delay, 10-year delay, and 20-year delay. Agricultural land value is inclusive of the social cost of nitrogen (SCN). Forest harvest is the sum of the clearcut and selection harvest values. Values are constant (2017) million USD and we use a 5% per annum discount rate for both intervention cost and carbon benefits..

	Climate Future			
	CanESM2	CNRM-CM5	HadGEM2-ES	MIROC5
	**no intervention delay**			
**Direct Cost**				
Total	8370	8524	8322	8475
Annualized	545	554	541	551
**Indirect Cost**				
Agriculture	6523	6830	6025	5425
SCN	*312*	*347*	*306*	*370*
Developed	1416	1342	1211	1157
Forest Harvest	-74	-40	-86	-107
Clearcut	*-30*	*16*	*-51*	*-86*
Selection	*-44*	*-56*	*-35*	*-21*
Total	7865	8131	7150	6475
Annualized	512	529	465	421
**Direct + Indirect Cost**				
Total	16236	16655	15472	14951
Annualized	1056	1083	1006	973
**Carbon Benefit**				
Total	4330	4334	5026	4267
Annualized	282	282	327	278
	**10-year intervention delay**			
**Direct Cost**				
Total	3506	3584	3541	3469
Annualized	281	288	284	278
**Indirect Cost**				
Agriculture	2208	1686	1396	1259
SCN	*189*	*263*	*268*	*247*
Developed	1441	1267	1207	1030
Forest Harvest	124	145	105	89
Clearcut	*254*	*288*	*208*	*184*
Selection	*-129*	*-143*	*-103*	*-95*
Total	3773	3098	2708	2378
Annualized	303	249	217	191
**Direct + indirect Cost**				
Total	7279	6681	6250	5846
Annualized	584	536	501	469
**Carbon Benefit**				
Total	4576	4638	3532	4979
Annualized	367	372	283	400
	**20-year intervention delay**			
**Direct Cost**				
Total	261	288	279	273
Annualized	34	37	36	35
**Indirect Cost**				
Agriculture	282	238	318	242
SCN	*132*	*136*	*123*	*123*
Developed	1433	1147	1257	1113

Forest Harvest	45	56	48	24
Clearcut	*100*	*128*	*108*	*61*
Selection	*-56*	*-72*	*-60*	*-37*
Total	1760	1441	1623	1379
Annualized	228	187	210	179
**Direct + Indirect Cost**				
Total	2021	1728	1902	1652
Annualized	262	224	246	214
**Carbon Benefit**				
Total	3703	4292	4291	5015
Annualized	480	556	556	649

Table S3.

Estimated maximum values and costs of NCS interventions. NCS interventions relative to the no intervention scenario over the period 2020-2050, for no delay, 10-year delay, and 20-year delay. Agricultural land value is inclusive of the social cost of nitrogen (SCN). Forest harvest is the sum of the clearcut and selection harvest values. Values are constant (2017) million USD and we use a 5% per annum discount rate for both intervention cost and carbon benefits.

	Climate Future			
	CanESM2	CNRM-CM5	HadGEM2-ES	MIROC5
**Direct Cost**				
Total	5872	5850	5879	5876
Annualized	382	381	382	382
**Indirect Cost**				
Agriculture	5048	5344	4959	5036
SCN	*-7*	*3*	*-11*	*-12*
Developed	838	863	850	804
Forest Harvest	-126	-113	-131	-153
Clearcut	*-76*	*-49*	*-89*	*-121*
Selection	*-50*	*-64*	*-42*	*-33*
Total	5760	6094	5678	5686
Annualized	375	396	369	370
**Direct + Indirect Costs**				
Total	11632	11944	11557	11562
Annualized	757	777	752	752
**Carbon Benefit**				
Total	9842	10501	9582	9846
Annualized	470	502	458	470

Table S4.

Economic costs and benefits from 2020 NCS scenario using a 5% discount rate for intervention costs but a 2.5% discount rate for carbon benefits. Estimated direct and indirect costs and benefits of NCS interventions relative to the no intervention scenario over the period 2020-2050. Agricultural land value is inclusive of the social cost of nitrogen (SCN). Forest harvest is the sum of the clearcut and selection harvest values. Values are constant (2017) million USD and we use a 2.5% per annum discount rate for carbon benefits and a 5% per annum discount rate for intervention costs.

	Climate Future											
	CanESM2			CNRM-CM5			HadGEM2-ES			MIROC5		
	cost		benefit	cost		benefit	cost		benefit	cost		benefit
**Intervention**	direct	indirect		direct	indirect		direct	indirect		direct	indirect	
Reforestation	36.06	—	48.82	no seq	—	no seq	94.87	—	-40.75	46.02	—	44.05
CFM	—	-2.12	54.60	—	-1.93	55.15	—	-2.08	51.69	—	-2.35	55.73
Agroforestry	5.78	—	45.43	no seq	—	no seq	no seq	—	no seq	3.82	—	50.09
Cover Crop	710.15	—	83.06	1,335.09	—	77.70	403.01	—	28.97	162.33	—	59.53
Soil Amend.	38.82	—	89.88	no seq	—	no seq	16.35	—	50.39	32.13	—	55.99
Conservation	—	69.86	51.96	—	77.00	50.89	—	71.78	49.02	—	63.75	52.43
Combined	32.64	32.02	54.71	30.27	31.53	54.34	32.14	31.04	52.38	31.79	30.76	53.27

Table S5.

Cost and benefit per ton of CO_2e for individual and combined interventions using a 5% discount rate for intervention costs but a 2.5% discount rate for carbon benefits. Direct and indirect costs and carbon benefit per ton of CO_2e sequestered relative to the no intervention scenario over the period 2020-2050. Interventions marked “no seq” means no net carbon storage under that climate future. Aggregate costs and benefits for each intervention are shown in Table S5. Dashes indicate no cost associated with the intervention. Values are constant (2017) USD and we use a 2.5% per annum discount rate for carbon benefits and a 5% per annum discount rate for intervention costs.

	Climate Future			
	CanESM2	CNRM-CM5	HadGEM2-ES	MIROC5
	**10-year intervention delay**			
**Direct Cost**				
Total	2343	2344	2342	2337
Annualized	188	188	188	188
**Indirect Cost**				
Agriculture	1475	1671	1342	1466
SCN	*27*	*38*	*37*	*27*
Developed	840	855	835	777
Forest Harvest	79	100	56	41
Clearcut	*212*	*247*	*169*	*150*
Selection	*-132*	*-147*	*-112*	*-109*
Total	2394	2627	2234	2284
Annualized	192	211	179	183
**Direct + Indirect Cost**				
Total	4737	4971	4575	4621
Annualized	380	399	367	371
**Carbon Benefit**				
Total	870	1000	813	937
Annualized	94	99	89	92
	**20-year intervention delay**			
**Direct Cost**				
Total	626	625	631	623
Annualized	81	81	82	81
**Indirect Cost**				
Agriculture	247	205	249	220
SCN	*21*	*21*	*22*	*21*
Developed	834	824	844	784
Forest Harvest	28	41	32	6
Clearcut	*89*	*115*	*94*	*51*
Selection	*-61*	*-74*	*-62*	*-45*
Total	1108	1070	1125	1009
Annualized	144	139	146	131
**Direct + Indirect Cost**				
Total	1735	1694	1756	1632
Annualized	225	219	227	211
**Carbon Benefit**				
Total	641	695	661	767
Annualized	83	90	86	99

Table S6.

Economic results from delayed NCS scenarios. Estimated values and costs of delayed NCS interventions relative to the no intervention scenario over the period 2020-2050. Agricultural land value is inclusive of the social cost of nitrogen (SCN). Forest harvest is the sum of the clearcut and selection harvest values. Values are constant (2017) million USD and we use a 5% per annum discount rate for both intervention cost and carbon benefits.

	Climate Future			
	CanESM2	CNRM-CM5	HadGEM2-ES	MIROC5
**Direct Costs**				
Reforestation	452	441	462	453
CFM	—	—	—	—
Agroforestry	58	57	58	58
Cover Crop	5064	5055	5062	5066
Soil Amendments	298	297	298	298
Conservation	—	—	—	—
**Indirect Costs**				
CFM	-126	-113	-131	-153
Conservation	5886	6207	5809	5839
**Carbon Benefit**				
Reforestation	61.0	-113.4	-71.3	23.5
CFM	497.8	492.5	483.5	548.6
Agroforestry	58.4	-60.3	-43.2	95.5
Cover Crop	100.8	34.4	30.1	281.6
Soil Amendments	120.6	-27.5	130.6	62.3
Conservation	653.1	595.6	584.2	699.5

Table S7.

Economic costs and benefits from individual 2020 NCS scenarios. Estimated indirect and direct costs and carbon benefit of NCS interventions relative to the no intervention scenario over the period 2020-2050. Values are constant (2017) million USD and we use a 5% per annum discount rate for both intervention cost and carbon benefits..

Year	Discount rate 5%	3%	2.5%
2020	14.50	50.77	74.94
2025	16.92	55.60	82.19
2030	19.34	60.44	88.24

Table S8.

The social cost of a metric ton of CO_2 (SC-CO_2) in California (2017 USD). Values from Table 7 in ref. [61] given in 2007 USD. We used CCPI-U = California Consumer Price Index - All Urban Consumers to convert these to the 2017 USD values shown here.

Year	Discount rate 5%	3%	2.5%
2020	53.18	186.14	274.78
2025	62.05	203.87	301.37
2030	70.91	221.60	323.53

Table S9:

The social cost of a metric ton of C in California (2017 USD). Values from Table 7 in ref. [61] given in 2007 USD and converted from metric tons of CO_2 to metric tons of carbon (C).

	Range in estimates of $ per acre (2017 USD)					
**Intervention**	**Lowest**	**Low**	**Middle**	**High**	**Highest**	**Mean**
Cover crop	84.00	89.50	95.00	98.00	101.00	93.50
Agroforestry	85.57	129.40	288.02	486.30	1277.31	453.32
Reforestation	241.00	530.00	706.00	878.00	1000.00	671.00
Soil amendments	300	300	300	300	300	300

Table S10.

Annual per acre costs (2017 USD) for the interventions. Sources of data: Cover cropping[75]; agroforestry[75]; reforestation[75]; soil amendments[76]

County	Orchard	Vineyard	Annual	Irrigated Pasture	Dry Pasture	Rangeland (low)	Rangeland (high)
Alameda	3483.61	2850.39	1640.69	229.28	19.00	5.21	31.24
Alpine	6286.09	546.02	1639.50	139.30	18.85	7.90	18.43
Amador	3883.11	699.24	343.20	94.91	32.19	9.10	40.34
Butte	2810.11	0.00	440.28	131.71	19.05	10.41	27.57
Calaveras	4056.98	1172.16	127.74	143.01	19.43	7.82	32.16
Colusa	3235.95	3375.26	479.76	155.89	18.85	10.41	27.57
Contra Costa	2336.86	1192.22	726.64	305.10	26.37	7.78	39.02
Del Norte	0.00	0.00	557.84	218.48	64.79	0.00	0.00
El Dorado	6286.09	392.79	39.26	148.40	18.97	10.41	41.65
Fresno	2051.32	1423.76	1168.17	135.77	15.10	5.21	31.24
Glenn	2412.80	3559.38	334.55	181.97	9.57	10.41	27.57
Humboldt	1712.27	2596.67	289.28	145.03	23.26	0.00	0.00
Imperial	1641.85	5174.64	580.69	1097.58	4.57	0.00	0.00
Inyo	2027.05	6530.12	827.89	58.37	3.21	7.90	18.43
Kern	2659.88	10399.03	740.81	147.94	14.95	6.76	24.45
Kings	3176.71	2441.12	608.53	225.10	5.94	6.76	24.45
Lake	1954.13	4522.08	284.30	140.04	8.19	10.41	32.26
Lassen	1268.51	0.00	131.01	179.69	1.05	7.90	18.43
Los Angeles	2468.73	10399.03	635.28	136.51	24.15	6.24	27.85
Madera	3790.71	1278.43	372.76	130.21	14.03	8.89	31.24
Marin	2182.82	1226.48	105.40	178.23	68.38	9.10	40.34
Mariposa	3990.62	1321.26	414.03	128.21	15.12	7.90	18.43
Mendocino	1712.27	2596.67	100.86	144.87	10.61	10.41	27.57
Merced	3995.43	1168.61	592.60	198.66	25.11	7.78	39.02
Modoc	1268.51	0.00	296.31	120.53	13.81	0.00	0.00
Mono	2921.01	1351.09	1060.14	60.97	4.19	7.90	18.43
Monterey	4404.79	1756.32	2639.70	0.00	17.39	6.25	31.24
Napa	1795.99	12696.95	145.70	120.93	3.97	10.41	41.65
Nevada	1957.01	831.18	137.61	185.70	20.05	10.41	41.65
Orange	1169.19	5174.64	226.60	777.22	8.41	0.00	0.00
Placer	2092.97	853.85	178.89	160.26	21.37	10.41	41.65
Plumas	1971.82	0.00	90.47	116.98	6.57	7.90	18.43
Riverside	1915.95	7510.51	735.45	141.98	2.11	0.00	0.00
Sacramento	1434.99	1337.58	226.63	149.58	26.70	10.41	41.65
San Benito	103.80	1934.49	2074.07	213.58	21.78	7.30	31.24
San Bernardino	223.88	8954.77	1032.37	136.51	0.33	7.33	21.44
San Diego	1367.75	2838.77	17542.16	2053.18	7.03	0.00	0.00
San Francisco	2336.86	1909.51	1859.65	230.69	32.28	6.07	33.83
San Joaquin	3928.25	1300.49	300.55	204.47	24.52	7.78	39.02
San Luis Obispo	306.54	2137.40	913.15	171.79	7.67	5.21	20.82
San Mateo	14644.21	2368.95	4746.90	156.28	15.38	5.21	31.24
Santa Barbara	1369.07	3188.29	958.13	171.79	9.63	7.29	20.82
Santa Clara	5732.29	1511.57	2152.64	228.50	13.51	18.74	31.24
Santa Cruz	14644.21	3679.42	2775.23	192.39	17.01	5.21	31.24
Shasta	1268.51	0.00	368.71	143.01	10.73	9.16	23.00
Sierra Valley	1821.05	831.18	123.24	116.98	6.57	9.16	30.04
Siskiyou	1268.51	0.00	557.84	109.90	9.07	0.00	0.00
Solano	2028.77	1059.40	114.23	124.61	22.42	10.41	41.65
Sonoma	2008.01	1794.59	16.48	104.97	17.91	9.10	40.34
Stanislaus	4185.71	1516.75	276.73	252.05	21.36	7.78	39.02
Sutter	1314.61	1783.01	254.29	176.67	19.61	10.41	41.65
Tehama	1987.61	2596.67	31.71	185.80	10.55	10.41	27.57
Trinity	1656.13	2596.67	20.71	106.72	8.55	10.41	27.57

Tulare	3173.15	7767.58	370.23	225.10	20.91	6.76	9.89
Tuolumne	3988.21	1397.59	604.18	132.99	19.74	7.90	18.43
Ventura	4522.44	6793.66	9607.33	171.79	0.32	5.72	31.24
Yolo	1405.07	2228.45	306.40	133.19	18.84	10.41	41.65
Yuba	1821.05	831.18	110.69	161.98	20.88	10.41	41.65

Table S11.
Annualized net return per acre to agriculture (2017 USD)

County	2007	2012	Average
Alameda	$4,274.19	$3,785.46	$4,029.83
Alpine	$6,438.66	$5,878.18	$6,158.42
Amador	$6,099.81	$3,309.26	$4,704.53
Butte	$2,314.78	$0.00	$1,157.39
Calaveras	$4,759.50	$3,285.35	$4,022.42
Colusa	$0.00	$770.18	$385.09
Contra Costa	$5,708.16	$6,764.58	$6,236.37
Del Norte	$1,133.20	$1,034.55	$1,083.87
El Dorado	$14,625.47	$9,893.79	$12,259.63
Fresno	$0.00	$0.00	$0.00
Glenn	$0.00	$0.00	$0.00
Humboldt	$2,050.21	$1,352.97	$1,701.59
Imperial	$0.00	$0.00	$0.00
Inyo	$834.61	$465.28	$649.94
Kern	$0.00	$0.00	$0.00
Kings	$0.00	$0.00	$0.00
Lake	$10,517.03	$2,660.31	$6,588.67
Lassen	$1,392.98	$2,057.18	$1,725.08
Los Angeles	$6,300.02	$14,781.80	$10,540.91
Madera	$0.00	$0.00	$0.00
Marin	$4,242.57	$5,721.61	$4,982.09
Mariposa	$2,107.27	$2,172.59	$2,139.93
Mendocino	$6,348.41	$3,767.87	$5,058.14
Merced	$0.00	$0.00	$0.00
Modoc	$1,181.19	$1,241.25	$1,211.22
Mono	$1,961.45	$0.00	$980.73
Monterey	$0.00	$0.00	$0.00
Napa	$28,304.35	$17,599.64	$22,951.99
Nevada	$11,295.77	$15,147.95	$13,221.86
Orange	$0.00	$32,049.11	$16,024.56
Placer	$12,972.63	$12,876.35	$12,924.49
Plumas	$2,209.70	$1,945.51	$2,077.60
Riverside	$4,099.55	$2,854.60	$3,477.07
Sacramento	$1,315.01	$4,480.48	$2,897.74
San Benito	$872.27	$1,831.92	$1,352.10
San Bernardino	$0.00	$0.00	$0.00
San Diego	$7,062.36	$11,681.11	$9,371.73
San Francisco	$0.00	$0.00	$0.00
San Joaquin	$0.00	$0.00	$0.00
San Luis Obispo	$4,154.58	$2,781.35	$3,467.96
San Mateo	$0.00	$16,845.08	$8,422.54
Santa Barbara	$2,789.34	$1,319.24	$2,054.29
Santa Clara	$4,276.15	$3,814.69	$4,045.42
Santa Cruz	$0.00	$0.00	$0.00
Shasta	$4,194.12	$2,706.17	$3,450.15

Sierra Valley	$3,032.84	$570.27	$1,801.55
Siskiyou	$2,845.93	$1,957.58	$2,401.76
Solano	$1,858.59	$3,249.47	$2,554.03
Sonoma	$15,509.60	$8,697.32	$12,103.46
Stanislaus	$0.00	$0.00	$0.00
Sutter	$1,855.89	$0.00	$927.94
Tehama	$2,677.50	$1,749.55	$2,213.52
Trinity	$1,700.84	$1,552.78	$1,626.81
Tulare	$0.00	$0.00	$0.00
Tuolumne	$4,560.59	$6,152.69	$5,356.64
Ventura	$0.00	$0.00	$0.00
Yolo	$1,652.73	$0.00	$826.37
Yuba	$3,686.43	$1,061.67	$2,374.05

Table S12.

Value of a development right for years 2007 and 2012. Values are per-acre inflation-adjusted VDR (2017 USD).

County	Net return to development
Alameda	201.49
Alpine	307.92
Amador	235.23
Butte	57.87
Calaveras	201.12
Colusa	19.25
Contra Costa	311.82
Del Norte	54.19
El Dorado	612.98
Fresno	49.71
Glenn	154.03
Humboldt	85.08
Imperial	321.22
Inyo	32.50
Kern	208.91
Kings	173.40
Lake	329.43
Lassen	86.25
Los Angeles	527.05
Madera	141.29
Marin	249.10
Mariposa	107.00
Mendocino	252.91
Merced	125.62
Modoc	60.56
Mono	49.04
Monterey	120.50
Napa	1147.60
Nevada	661.09
Orange	801.23
Placer	646.22
Plumas	103.88
Riverside	173.85
Sacramento	144.89
San Benito	67.60
San Bernardino	383.66
San Diego	468.59
San Francisco	295.89
San Joaquin	218.91
San Luis Obispo	173.40
San Mateo	421.13
Santa Barbara	102.71
Santa Clara	202.27
Santa Cruz	230.33
Shasta	172.51

Sierra Valley	90.08
Siskiyou	120.09
Solano	127.70
Sonoma	605.17
Stanislaus	195.94
Sutter	46.40
Tehama	110.68
Trinity	81.34
Tulare	32.50
Tuolumne	267.83
Ventura	314.88
Yolo	41.32
Yuba	118.70

Table S13.

Annualized per acre value of a development right circa 2010. Values are net return to development in 2017 USD.

County	Orchard	Vineyard	Annual	Irr Pasture	Dry Pasture	Rangeland
Alameda	128.46	19.50	70.14	41.33	0.00	0.00
Alpine	129.37	5.00	80.00	88.00	0.00	0.00
Amador	172.25	5.00	54.29	88.00	0.00	0.00
Butte	172.85	20.00	182.18	41.33	0.00	0.00
Calaveras	162.07	5.00	80.00	88.00	0.00	0.00
Colusa	181.41	19.83	170.57	41.33	0.00	0.00
Contra Costa	97.68	19.50	179.52	41.33	0.00	0.00
Del Norte	0.00	0.00	80.00	88.00	0.00	0.00
El Dorado	53.78	5.00	80.00	88.00	0.00	0.00
Fresno	155.62	87.98	179.25	41.33	0.00	0.00
Glenn	141.68	19.50	160.16	41.33	0.00	0.00
Humboldt	123.50	12.25	80.00	88.00	0.00	0.00
Imperial	145.97	41.44	157.23	41.33	0.00	0.00
Inyo	137.46	49.97	146.10	41.33	0.00	0.00
Kern	154.38	40.63	119.52	41.33	0.00	0.00
Kings	160.08	34.75	180.06	41.33	0.00	0.00
Lake	148.01	19.50	148.19	56.89	0.00	0.00
Lassen	172.25	5.00	95.19	88.00	0.00	0.00
Los Angeles	125.77	45.75	41.66	41.33	0.00	0.00
Madera	165.35	33.71	143.41	41.33	0.00	0.00
Marin	99.44	19.50	113.99	56.89	0.00	0.00
Mariposa	168.82	29.25	129.84	41.33	0.00	0.00
Mendocino	123.50	19.50	97.27	64.67	0.00	0.00
Merced	171.69	29.25	148.21	41.33	0.00	0.00
Modoc	172.25	5.00	51.55	88.00	0.00	0.00
Mono	160.49	42.23	120.67	41.33	0.00	0.00
Monterey	175.00	29.25	154.30	0.00	0.00	0.00
Napa	129.25	19.50	116.43	56.89	0.00	0.00
Nevada	161.43	5.00	108.04	88.00	0.00	0.00
Orange	133.43	38.81	101.70	41.33	0.00	0.00
Placer	172.25	5.00	113.07	88.00	0.00	0.00
Plumas	160.75	5.00	31.20	88.00	0.00	0.00
Riverside	166.27	41.44	93.33	41.33	0.00	0.00
Sacramento	160.00	20.00	131.03	41.33	0.00	0.00
San Benito	115.07	29.25	134.29	41.33	0.00	0.00
San Bernardino	108.37	29.25	112.41	41.33	0.00	0.00
San Diego	125.66	41.44	121.98	41.33	0.00	0.00
San Francisco	136.34	19.50	130.54	41.33	0.00	0.00
San Joaquin	152.21	29.25	158.16	41.33	0.00	0.00
San Luis Obispo	136.56	29.25	109.29	41.33	0.00	0.00
San Mateo	175.00	19.50	158.49	41.33	0.00	0.00
Santa Barbara	127.07	29.25	137.91	41.33	0.00	0.00
Santa Clara	48.00	29.25	170.40	41.33	0.00	0.00
Santa Cruz	94.75	29.25	173.76	41.33	0.00	0.00
Shasta	172.25	5.00	35.50	88.00	0.00	0.00
Sierra Valley	150.61	5.00	30.92	88.00	0.00	0.00
Siskiyou	172.25	5.00	57.99	88.00	0.00	0.00
Solano	177.54	20.00	87.29	41.33	0.00	0.00
Sonoma	23.10	19.50	133.39	88.00	0.00	0.00
Stanislaus	169.43	29.25	147.75	41.33	0.00	0.00
Sutter	154.03	20.00	171.24	41.33	0.00	0.00
Tehama	147.29	9.83	70.82	41.33	0.00	0.00
Trinity	147.68	5.00	42.00	88.00	0.00	0.00

Tulare	131.48	42.02	173.20	41.33	0.00	0.00
Tuolumne	165.62	20.44	80.00	41.33	0.00	0.00
Ventura	114.56	38.54	195.75	41.33	0.00	0.00
Yolo	168.34	20.00	128.62	41.33	0.00	0.00
Yuba	150.61	10.00	180.14	41.33	0.00	0.00

Table S14.

Average annual pounds of nitrogen applied to an acre of agriculture type. (N_{ik})
